# Computational optimization of 3D printed bone scaffolds using orthogonal array-driven FEA and neural network modeling

**DOI:** 10.1038/s41598-025-15122-5

**Published:** 2025-08-20

**Authors:** Amulya Shetty, Aamirah Fathima, B Anika, Raviraj Shetty, Vinyas, J.P. Supriya, Adithya Hegde

**Affiliations:** 1 Father Muller Medical College, Mangalore, Karnataka 575002 India; 2 Department of Mechanical and Industrial Engineering, Manipal Institute of Technology, Manipal Academy of Higher Education, Manipal, 576104 India

**Keywords:** Additively manufactured scaffolds, Bone tissue engineering, Taguchi design of experiments, Finite element analysis, Artificial neural network, Mechanical characterization, Microstructural analysis, Soft computing techniques, Biomedical engineering, Biomaterials, Structural materials

## Abstract

Today, orthopedic surgeons have been continuously focusing on bone tissue engineering for regenerating damaged bone through the use of biomimetic scaffolds and innovative materials. Hence, this study presents a comprehensive investigation into the optimization of PLA + 3D printed lattice scaffolds for bone tissue engineering applications, emphasizing the role of geometric configuration and processing parameters on mechanical performance. Three distinct lattice geometries such as Lidinoid, Diamond, and Gyroid were developed with varying wall thicknesses (1.0 mm, 1.5 mm, and 2.0 mm) and subjected to compressive loads of 3 kN, 6 kN, and 9 kN. A Taguchi L_27_ Orthogonal Array was employed to evaluate key mechanical responses, including displacement and strain. Among these configurations, the Gyroid lattice exhibited superior mechanical integrity, demonstrating the least displacement (0.36 mm) and strain (1.2 × 10⁻²) at 3 kN with 2.0 mm thickness, whereas the Lidinoid structure showed the highest deformability. A Back-propagation Artificial Neural Network (BPANN) model was developed to predict scaffold behavior with remarkable accuracy (R² = 0.9991 for displacement, R² = 0.9954 for strain), further Finite Element Analysis (FEA) was conducted to validate both experimental and predicted results. The novelty of this work lies in its integrative, multi-modal approach that synergizes experimental design, machine learning-based predictive modeling, and simulation. The focus of this study is to define a robust framework for optimizing scaffold architecture, with significant implications for enhancing mechanical strength and biological performance in bone healing applications.

## Introduction

Bone tissue engineering is a rapidly evolving and interdisciplinary field focused on the repair and regeneration of damaged bone through the development of biomimetic scaffolds and advanced materials. This domain integrates principles from cell biology, materials science, biomechanics, and clinical medicine to address critical challenges such as non-union fractures, large bone defects, and bone loss resulting from trauma, disease, or congenital anomalies. A key objective is to replicate the complex environment of native bone not only its biological and chemical cues that stimulate osteogenesis, but also its inherent mechanical strength and hierarchical architecture. Recent advances in stem cell technology, growth factor delivery, and gene therapy have further enriched scaffold design, enabling the development of dynamic constructs that can integrate with and adapt to the body’s natural healing mechanisms. Moreover, the incorporation of advanced imaging and computational modeling has enhanced scaffold design and performance prediction, ensuring that engineered tissues can better withstand physiological loads and effectively transfer stress during bone regeneration.

The advent of three-dimensional (3D) printing has revolutionized scaffold fabrication by enabling precise control over internal architecture. 3D-printed lattices offer a customizable platform where parameters such as pore size, shape, and interconnectivity can be finely tuned to influence cell behavior, nutrient transport, and vascularization. This capability facilitates the creation of complex structures that closely mimic the trabecular architecture of natural bone, thereby enhancing both mechanical stability and biological integration. The technology supports a broad range of biocompatible materials including bioactive ceramics, polymers, and metal alloys broadening the application spectrum of 3D-printed scaffolds. Furthermore, layer-by-layer deposition enables the fabrication of patient-specific constructs derived from medical imaging data, ensuring a precise match between scaffold geometry and the bone defect site. Innovations in multi-material printing have also made it possible to produce gradient scaffolds, wherein material composition varies spatially to replicate the transition from cortical to cancellous bone.

Various geometric configurations of 3D-printed lattices have been investigated for their impact on scaffold mechanics and biological performance. Cubic lattices, known for their straightforward yet robust framework, provide uniform stress distribution and considerable compressive strength, while maintaining adequate porosity for cellular infiltration. In contrast, hexagonal lattices offer enhanced interconnectivity between struts, which improves force distribution and reduces the likelihood of stress concentration-induced failure.

More intricate geometries such as gyroid and diamond structures have gained considerable attention due to their continuous, non-self-intersecting networks. These structures exhibit superior resistance to deformation and maximize internal surface area attributes conducive to cell adhesion and proliferation. Studies have shown that variations in strut thickness, pore size, and overall connectivity directly affect a scaffold’s stiffness, yield strength, and fatigue life. For instance, increasing strut thickness can enhance load-bearing capacity, but often at the cost of reduced porosity, which may hinder nutrient diffusion and cell migration. Conversely, highly porous designs may improve biological functionality but compromise mechanical integrity. Balancing these trade-offs is critical and is continually refined through experimental research and computational simulations aimed at achieving optimal scaffold performance for clinical use.

Recent developments in additive manufacturing have enabled the creation of highly complex scaffold geometries that are challenging to produce via conventional methods. This technological capability has advanced the design of architected materials commonly referred to as metamaterials featuring periodic unit cell repetition in three dimensions. Such structures exhibit mechanical properties superior to conventional designs, making them ideal for lightweight and high-performance applications across sectors such as aerospace, automotive, and biomedicine^1^.

Lattice structures have shown promise in applications requiring compressive strength and controlled deformation, including energy absorption in aerospace, damping systems in civil infrastructure, and load distribution in orthopedic implants^2^. Their geometric intricacy necessitates the use of additive manufacturing, as traditional techniques fall short in fabricating such detailed architectures. Multiple unit cell types including Kelvin cells, gyroids, and other triply periodic minimal surface (TPMS) structures have been studied for their unique mechanical responses under various loading regimes^3,4^.

The mechanical performance of 3D-printed lattice structures is governed by both design parameters and processing conditions. Factors such as porosity, strut thickness, cell geometry, and material composition significantly influence compressive behavior^5^. For example, research on 389 distinct gyroid lattices fabricated via vat photopolymerization has revealed intricate relationships between material selection, porosity levels, and mechanical outcomes^6^. Similarly, studies on thermoplastic polyurethane (TPU) lattices with Kelvin configurations have demonstrated differing responses between uniform and spatially graded structures under static and dynamic loads^7^.

Standardized compressive testing protocols often following ASTM guidelines are used to evaluate the mechanical performance of such structures. Tests are typically conducted using devices like the MTS Criterion C43.504 system^8^, with samples (usually cylindrical, 12 mm in diameter and 17 mm in height) subjected to displacement-controlled loading at rates such as 5 mm/min until failure^9^. Force-displacement data is normalized to derive stress-strain relationships, allowing comparison across designs. Key properties include elastic modulus, yield and ultimate compressive strength, and energy absorption capacity. Metrics like force-to-mass ratio (N/g) and force-to-area ratio (MPa) provide normalized evaluations of mechanical efficiency^10^. Reported compressive strengths typically range from 0.5 to 15 MPa, with failure strains between 5% and 30%, depending on design and material^11,12^.

Finite element analysis (FEA) has become indispensable in predicting the mechanical behavior of lattice structures. It complements experimental testing by offering insights into internal stress distributions and allows for rapid iteration of designs. Both static and dynamic FEA solvers, such as Abaqus/Standard and Abaqus/Explicit, are employed based on loading conditions^13,14^.

Accurate material modeling is essential for realistic simulation outcomes. For elastomeric lattices, hyperelastic models like the third-order Ogden formulation have shown good agreement with experiments^1^. Mesh convergence studies are generally performed to balance computation time with solution precision. For instance, simulations extending to 2 mm displacement (approx. 8% strain) are used to capture the linear elastic regime^15^. Maximum stress predictions from FEA help identify critical failure regions, with values often ranging from 10 to 50 MPa depending on structure and load^16^.

Recent trends emphasize optimizing scaffold performance via statistical and computational methods. Design of Experiments (DoE) approaches, such as the Doehlert Design combined with Response Surface Methodology (RSM), have proven efficient for tuning parameters in fused filament fabrication (FFF), where poor interlayer adhesion can result in anisotropic behavior and premature failure^17,18^.

Material-specific processing characteristics also affect performance. For instance, the rheological behavior of PLA and TPU during extrusion plays a key role in determining final mechanical strength^19^. The use of machine learning to model relationships between geometric features, process parameters, and resulting properties has added a predictive dimension to scaffold design^20^.

This study presents a detailed investigation into the mechanical performance and optimization of PLA + 3D-printed lattice scaffolds for bone tissue engineering. Three TPMS-based geometries Lidinoid, Diamond, and Gyroid are analyzed across three wall thicknesses (1.0 mm, 1.5 mm, and 2.0 mm) and under three compressive load levels (3 kN, 6 kN, and 9 kN). Key mechanical responses including displacement (µm) and strain are evaluated.

The methodology integrates experimental testing, computational modeling, and machine learning. A Taguchi L_27_ orthogonal array guides experimental design to assess multifactorial influences systematically. This design approach minimizes trial numbers while preserving statistical rigor.

The primary goal is to establish functional relationships between scaffold geometry, material configuration, and mechanical loading to optimize performance for clinical applications. A Backpropagation Artificial Neural Network (BPANN) is trained on empirical data to predict mechanical responses across a wider parametric space^21,22^.

The novelty of this study lies in its unified methodology, combining Taguchi-based experimental design, neural network prediction, and FEA validation. The FEA simulations substantiate the experimental and predicted results, providing a holistic understanding of mechanical behavior under physiologically relevant loading. This integrated approach forms a robust and scalable framework for designing mechanically efficient, biologically compatible bone scaffolds.

## Methodology

### Processing

In this study, the fabrication of bone scaffolds was carried out using additive manufacturing techniques to ensure precision, repeatability, and customizability in geometric configurations. PLA+ (Polylactic Acid Plus) was selected as the scaffold material owing to its enhanced mechanical properties, biocompatibility, and biodegradability, making it highly suitable for bone tissue engineering applications. Compared to standard PLA, PLA + offers improved toughness and reduced brittleness, which is beneficial in mimicking the mechanical behavior of cancellous bone while maintaining ease of printability.

The scaffold designs were created using nTop software, within a fixed envelope size of 30 × 30 × 30 mm³. The wall (strut) thickness was varied at three levels: 1.0 mm, 1.5 mm, and 2.0 mm.

The porosity for each structure was calculated based on the solid-to-total volume ratio using the parametric models in nTop. At 1.0 mm wall thickness, porosity was approximately 84% for Lidinoid, 83.5% for Diamond, and 85% for Gyroid. At 1.5 mm thickness, it was 77.2% (Lidinoid), 76.8% (Diamond), and 78.1% (Gyroid). At 2.0 mm thickness, the values reduced to 70.5% (Lidinoid), 69.8% (Diamond), and 71.3% (Gyroid).

The average pore size for all three lattices was maintained in the range of 700–900 μm, measured between opposite TPMS surfaces. Due to the nature of TPMS structures, pore sizes are not uniform but continuous and interconnected, promoting vascularization. The internal unit cell size was implicitly set by the number of periodic repetitions across the 30 mm cube and matched for all three geometries to maintain structural equivalence.

The material used for both fabrication and simulation of the scaffolds was PLA+ (Polylactic Acid Plus). For Finite Element Analysis, the following mechanical properties were used based on datasheets and literature: Young’s Modulus = 3.5 GPa, Poisson’s Ratio = 0.36, Yield Strength = 60 MPa, Ultimate Tensile Strength = 65 MPa, and Density = 1.24 g/cm³. These properties were assumed to be isotropic and homogeneous throughout the structure. A linear elastic material model was applied for the simulations, as the objective was to analyse the initial deformation response and compare structural stiffness across designs. While PLA + exhibits nonlinear plastic behaviour beyond the yield point, the applied loads in this study were selected such that the majority of deformation occurred within or near the elastic range. The model predictions showed close agreement with experimental results, validating the linear elastic assumption. In future work, incorporation of elastic–plastic material modelling will be considered for more accurate prediction of post-yield deformation under higher loads.

**Fig. 1:**
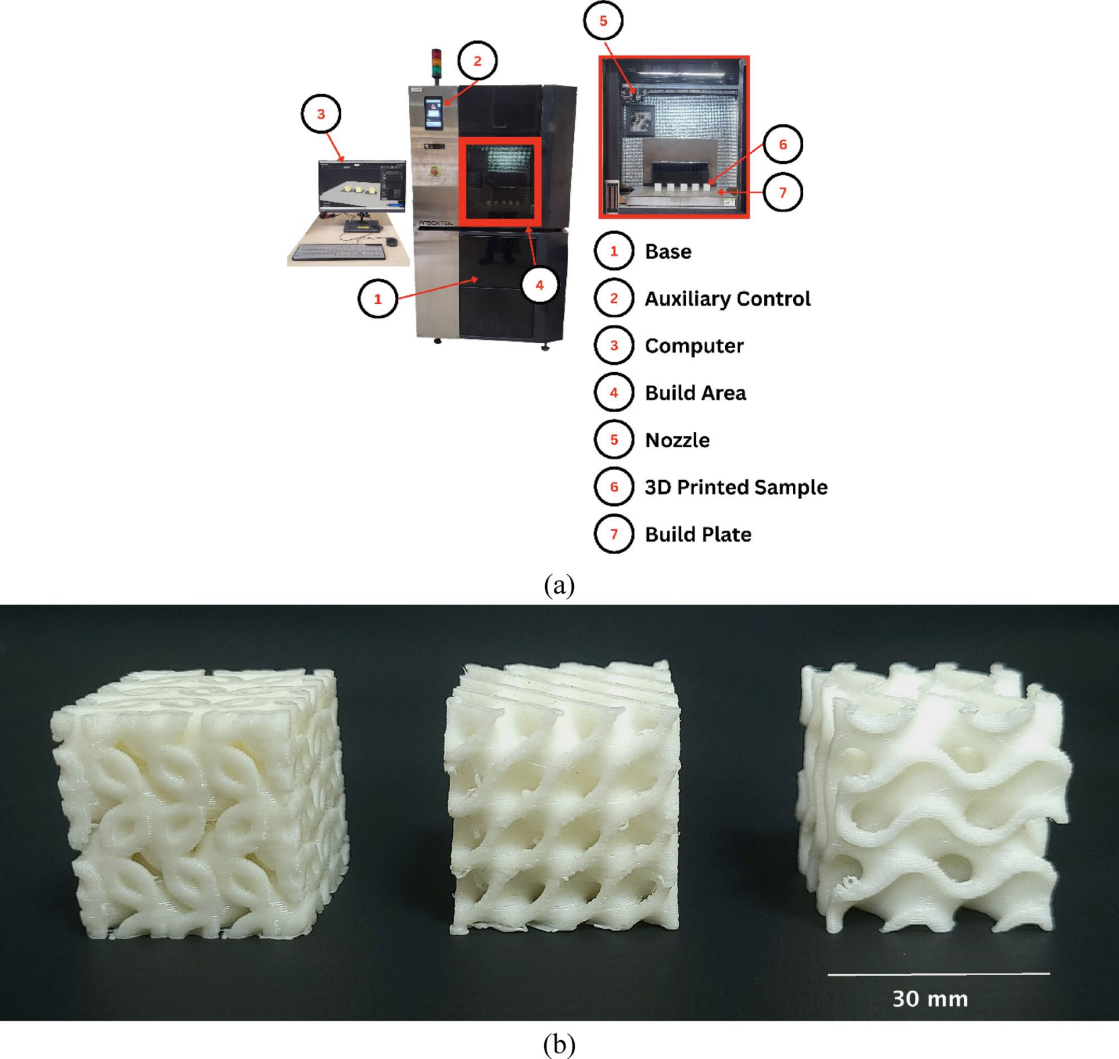
(**a**) Fracktel Volterra 3D printing setup; (**b**) 3D printed samples.

A ‘Fracktel Volterra FDM 3D printer’ (Fig. 1) was employed for scaffold fabrication, chosen for its high resolution and consistency in producing complex lattice structures. The scaffolds were designed with three distinct triply periodic minimal surface (TPMS) geometries: Lidinoid, Gyroid, and Diamond, each constrained within a 30 × 30 × 30 mm³ cubic envelope (Fig. 1). This dimension was selected to simulate a representative bone defect size commonly encountered in clinical segmental bone loss cases, allowing for a fair comparison between geometrical types. The selected TPMS structures are well-researched for their superior load-bearing ability, high surface-to-volume ratio, and interconnected porosity, all of which are essential for vascularization and osteointegration.

Wall thickness was varied across three levels i.e. 1.0 m, 1.5 mm, and 2.0 mm, to study its influence on the mechanical performance and porosity of the scaffolds. Thinner walls increase porosity and surface area beneficial for cell proliferation, while thicker walls offer higher mechanical strength, enabling the identification of an optimal balance between biological and structural requirements. The constant parameters maintained during the printing process, such as print speed, layer height, and nozzle temperature, etc. are detailed in Table 1, ensuring consistency across all experimental conditions. Print fidelity was verified by overlaying the STL geometry with the actual scaffold cross-section and capturing SEM micrographs to ensure resolution adequacy. Despite some fused segments, porosity was maintained and verified across all structures.

**Table 1:** 3D**-**Printing properties.

Parameter	Value
Layer Height	0.3 mm
Line Width	0.6 mm
Brim Width	0.6 mm
Skin Overlap Percentage	10%
In-fill Density	100%
In-fill Line distance	1.2 mm
In-fill pattern	Grid
Printing Temperature	210 °C
Build Plate Temperature	90 °C
Print Speed	30 mm/s
Wall Speed	22 m/s
Travel Speed	120 mm/s
Retraction Speed	60 mm/s
Brim Width	150 mm
Brim Line count	6

### Characterization

**Fig. 2:**
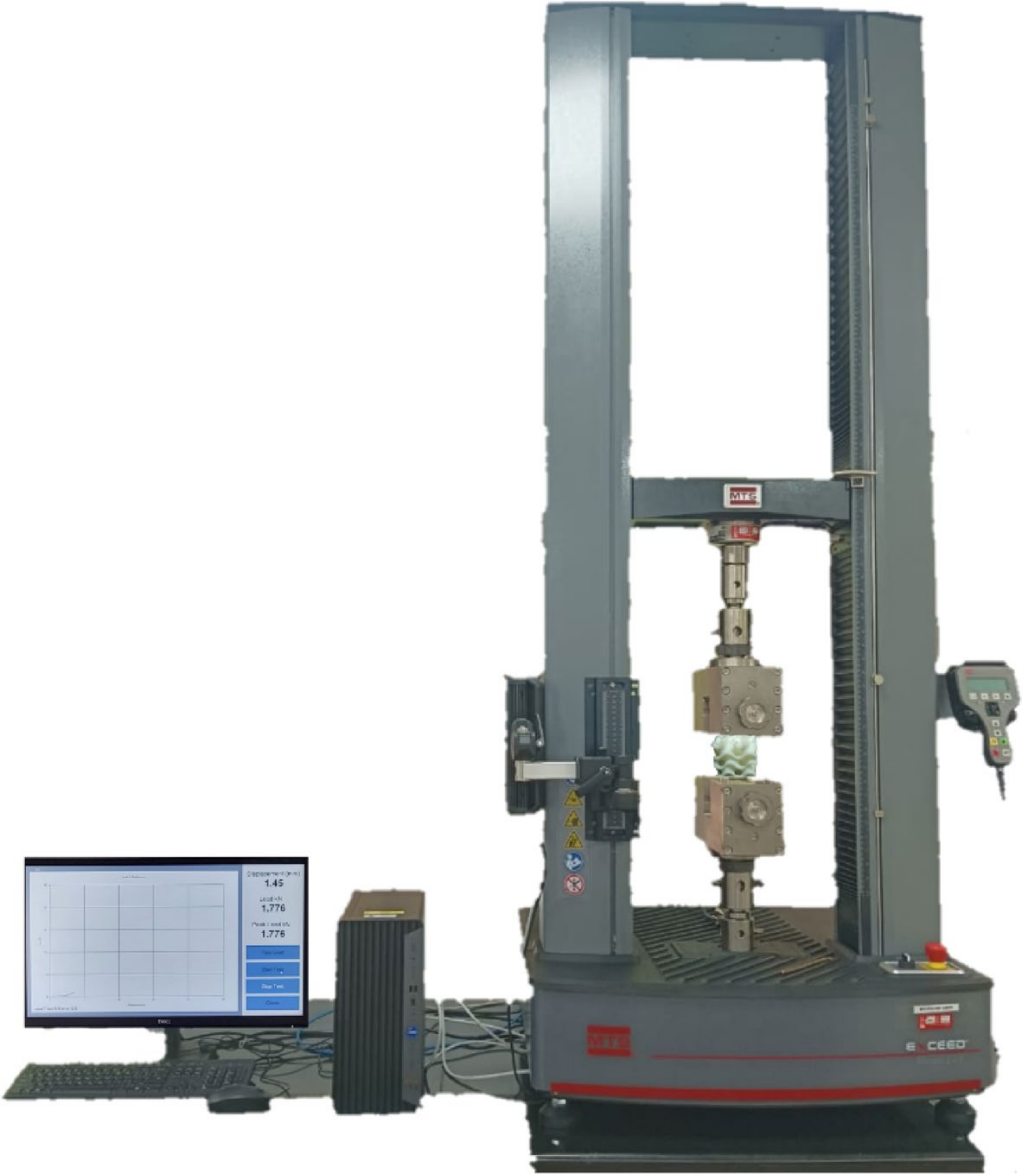
Universal testing machine.

To assess the mechanical performance of the 3D-printed bone scaffolds under physiological loading conditions, uniaxial compressive tests were conducted using a calibrated Universal Testing Machine (UTM) (Fig. 2). The scaffolds were subjected to three levels of axial compressive loads of 3 kN, 6 kN, and 9 kN. This load range was chosen to avoid premature structural failure while still provoking sufficient material response for evaluation.

During testing, the displacement was directly recorded using the built-in digital displacement transducer of the universal testing machine, which continuously monitored the compression of the scaffold along the axial direction. The strain was calculated by dividing the measured axial displacement by the original height of the scaffold (30 mm), based on the standard engineering strain formula provided in Eq. 1.

1 $$\:\varvec{S}\varvec{t}\varvec{r}\varvec{a}\varvec{i}\varvec{n}=\:\frac{\varvec{\Delta\:}\varvec{L}}{{\varvec{L}}_{0}}$$

Where, ΔL’ is the change in length (displacement) and L_0_ is the original length of the specimen.

### Design of experiments

To systematically investigate the influence of geometrical structure and wall thickness on the mechanical performance of 3D-printed bone scaffolds under varying compressive loads, a Design of Experiments (DoE) approach was adopted. Specifically, Taguchi’s L_27_ orthogonal array was employed, which allows for efficient experimentation with multiple factors and levels while minimizing the number of trials. The L_27_ array is suitable for handling three factors at three levels each, ensuring balanced representation and orthogonality among factor combinations. The selected control factors for this study are presented in Table 2. This experimental design enabled the analysis of main effects, interaction effects, and higher-order interactions among the parameters, thereby offering comprehensive insight into how each factor, as well as their combinations, affect the response variables.

For statistical evaluation, Analysis of Variance (ANOVA) was performed to determine the level of significance and contribution of each input factor to the output responses. ANOVA helped quantify the percentage contribution of each parameter to the variation observed in the experimental data, allowing the identification of dominant factors affecting scaffold performance.

All the statistical analyses and graphical visualizations were performed using *Minitab 15* software, which offered a comprehensive suite of tools for interpreting the experimental results obtained from the Taguchi design. A series of plots were generated to enhance the understanding of the influence and interaction of input parameters on the mechanical responses of the 3D-printed bone scaffolds. The Main Effects Plot was utilized to display the direct impact of each individual factor i.e. scaffold geometry, wall thickness, and compressive load on the response variables, thereby helping identify trends and optimal levels. The Interaction Plot revealed how combinations of factors influenced the outcomes, highlighting any synergistic or antagonistic effects that may not be evident through main effects alone. Histograms were employed to analyze the frequency distribution and spread of the measured values such as displacement and strain ensuring that the data was statistically sound and normally distributed. Further, the Area Plot provided a visual representation of how the response values varied across levels of multiple factors simultaneously, aiding in identifying overlapping effects. Additionally, Contour Plots were generated to map the two-dimensional response surface across pairs of variables, enabling the visualization of critical zones of response variation. Finally, 3D Surface Plots were used to depict the tri-variate relationship among input parameters and their collective influence on each output response, offering a comprehensive spatial overview of the system’s behavior.

**Table 2:** Factors and levels used in the experimentation.

Factors	Levels		
	I	II	III
Geometry Type	Lidinoid	Diamond	Gyroid
Wall Thickness (mm)	1.0	1.5	2.0
Load Applied (kN)	3	6	9

### Artificial neural networks

The following methodology was adopted to develop and validate a Backpropagation Artificial Neural Network (BPANN) model for predicting Displacement and Strain in lattice structures under compression testing. Although load is not a design variable, it was included in the ANN to simulate varying mechanical environments. Future models will integrate porosity and curvature metrics as geometric features to better capture the morphometric complexity of TPMS structures. Parameters such as specific surface area, pore interconnectivity, and local curvature will be extracted via advanced morphometric analysis. These variables are expected to significantly improve prediction and optimization accuracy, enabling ANN-driven design decisions beyond just wall thickness and load considerations.

**Fig. 3:**
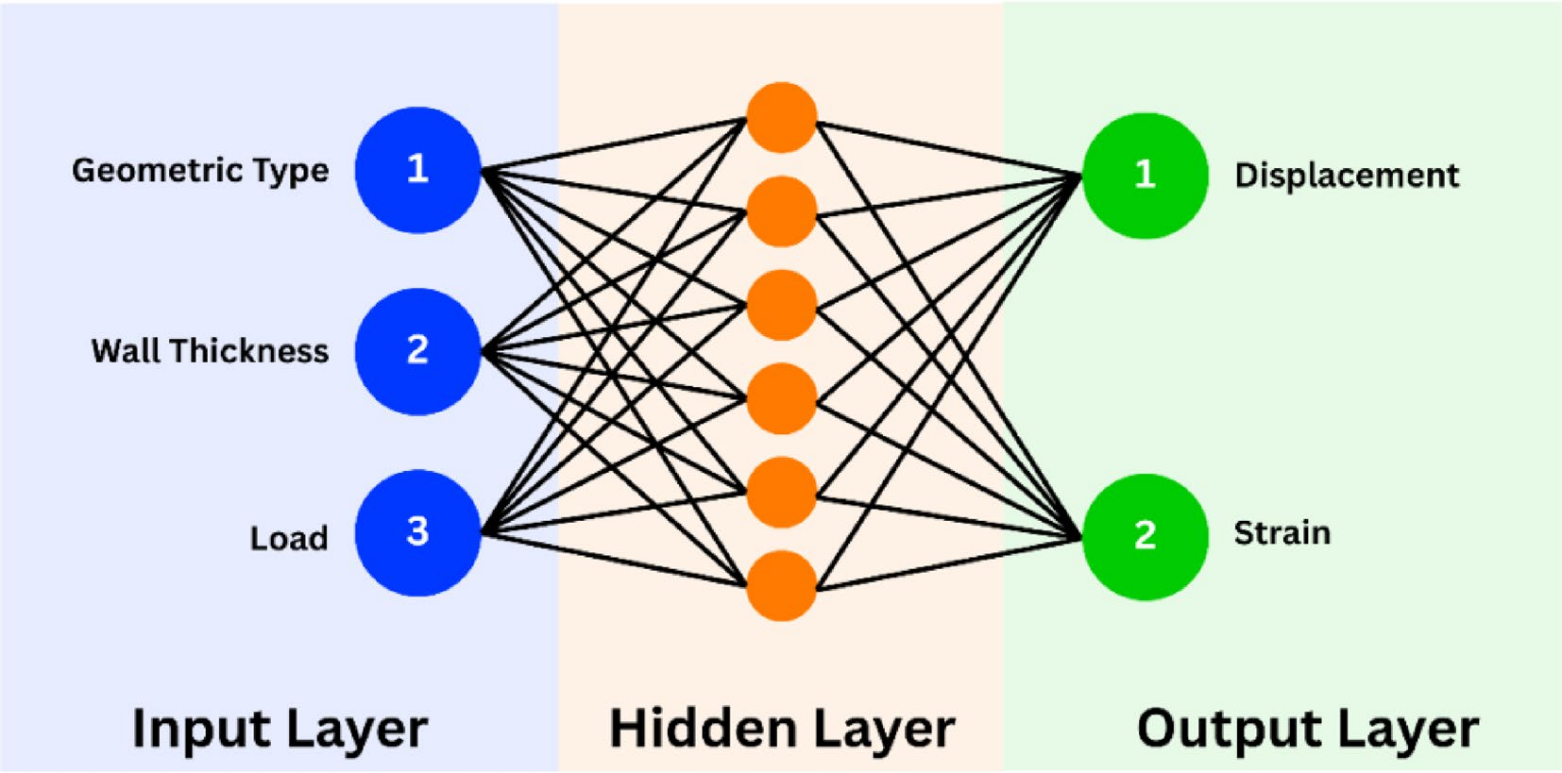
BPANN architecture.

1. ***Data Preparation and Feature Encoding***: The initial dataset comprised experimental and FEA-derived data from three lattice geometries—Lidinoid, Diamond, and Gyroid—subjected to various compressive loads. Input features included geometry type (categorical), wall thickness (mm), and applied load (N), while the outputs of interest were displacement (µm) and strain. Since neural networks require numerical inputs, the geometry type was transformed using one-hot encoding. This allowed the network to process categorical shape features without implying any ordinal relationship.
2. ***Dataset Division***: The complete dataset of 27 samples was divided into a training set (18 samples) and a testing set (9 samples). This 2:1 split ensured that the model was trained on a diverse yet representative subset of data while retaining enough unseen data to objectively evaluate its generalization capability.
3. ***Model Architecture and Training Setup***: A feed-forward Backpropagation Artificial Neural Network (BPANN) was implemented using a single hidden layer. The hidden layer consisted of 12 neurons, selected based on trial-and-error to balance model complexity and training stability. BPANN architecture has been presented in Fig. 3. Three activation functions i.e. ReLU, Tanh, and Sigmoid were compared to identify the most effective nonlinear mapping for this regression problem. The Adam optimizer was used with a learning rate of 0.01 for efficient gradient-based optimization, and the Mean Squared Error (MSE) was chosen as the loss function. The model was trained over 1000 epochs with a mini-batch size of 4 to ensure smooth convergence.
4. ***Model Evaluation and Activation Function Comparison***: To determine the best-performing activation function, the trained models were evaluated using standard regression metrics: Root Mean Square Error (RMSE), Mean Absolute Error (MAE), and R² (coefficient of determination). These metrics were calculated for each output (displacement and strain) individually and in aggregate. Additionally, Mean Absolute Percentage Error (MAPE) and maximum absolute error were included to gain a deeper understanding of prediction accuracy.

### Finite element analysis

This paper investigates the mechanical performance of three lattice structures (Lidinoid, Diamond, Gyroid) with varying wall thicknesses (1 mm, 1.5 mm, 2 mm) under different loading conditions (3 kN, 6 kN, 9 kN). The analysis was conducted using Finite Element Analysis (FEA) in nTop software. Each lattice structure had dimensions of 30 mm × 30 mm × 30 mm, and the wall thickness variations were incorporated to evaluate their effects on the mechanical properties.

The lattice structures were modeled by creating unit cells for each geometry (Lidinoid, Diamond, Gyroid). The unit cell geometry was parametrically defined to facilitate variations in lattice configurations. The strut radius for each lattice was calculated based on the wall thickness and the number of struts per unit cell, using the Eq. 2:

2 $$\:\varvec{r}=\frac{\varvec{t}}{2\varvec{n}}$$

where t is the wall thickness and n is the number of struts per unit cell. The equations used to define the lattice geometry were based on the parametric coordinates of the unit cell.

The Lidinoid lattice is generated by intersecting spheres within a unit cell. The position of each strut is defined by spherical coordinates, and the length of each strut depends on the lattice parameters. The equation for the coordinates of the struts in the Lidinoid lattice is given in Eq. 3.

3 $$\:\varvec{x}=\varvec{r}\mathbf{sin}\varvec{\theta\:}\mathbf{cos}\varvec{\upvarphi\:};\varvec{y}=\varvec{r}\mathbf{sin}\varvec{\theta\:}\mathbf{sin}\varvec{\upvarphi\:};\varvec{z}=\varvec{r}\mathbf{cos}\varvec{\theta\:}$$

where r is the radius of the sphere, θ is the polar angle, and ϕ is the azimuthal angle.

The Diamond lattice consists of four interconnected tetrahedra within a unit cell. The coordinates for the vertices of the tetrahedra can be expressed in Eq. 4.

4 $$\:\varvec{x}=\varvec{a}\mathbf{cos}\varvec{\theta\:};\varvec{y}=\varvec{a}\mathbf{sin}\varvec{\theta\:};\varvec{z}=\varvec{a}\mathbf{cos}\varvec\phi$$

where a is the lattice constant, and θ and ϕ are the angles defining the orientation of each tetrahedron.

The Gyroid lattice is defined by the intersection of three sinusoidal functions, and its structure is a minimal surface. The parametric equations to define the Gyroid lattice is given in Eq. 5.

5 $$\:\varvec{x}=\mathbf{sin}\varvec{k}\varvec{x}\times\:\mathbf{cos}\varvec{k}\varvec{y};\varvec{y}=\mathbf{sin}\varvec{k}\varvec{y}\times\:\mathbf{cos}\varvec{k}\varvec{z};\varvec{z}=\mathbf{sin}\varvec{k}\varvec{z}\times\:\mathbf{cos}\varvec{k}\varvec{x}$$

where k is the wave vector that defines the wavelength of the surface features, and x, y, z represent the spatial coordinates.

The lattice structures analyzed in this study were fabricated using PLA+ (Polylactic Acid Plus), a modified biopolymer widely used in 3D printing applications due to its improved toughness and mechanical strength compared to standard PLA. PLA + exhibits enhanced ductility and reduced brittleness, making it suitable for load-bearing structural components, especially in lattice-type geometries.

The top surface was modeled as a rigid plate to ensure uniform contact and simulate realistic compression. The bottom face was fully constrained (U1 = U2 = U3 = 0) to mimic fixed support conditions.

The mechanical properties of PLA + used for the finite element simulation were derived from standard datasheets provided by filament manufacturers and verified through literature. During the analysis, the Young’s Modulus (E) was considered as 3.5 GPa, followed by Poisson’s Ratio (ν) at 0.36, Yield Strength (σ_y_) at 60 MPa, Ultimate Tensile Strength (σ_uts_) at 65 MPa and Density (ρ) at 1.24 g/cm³. These properties were assumed to be isotropic and homogeneous throughout the lattice structures, which is a valid approximation for FEA in the absence of anisotropic behavior modeling. Since PLA + behaves as a linearly elastic material within its yield limit, a linear elastic material model was implemented in the simulation. This approach is appropriate considering the loading conditions (3 kN to 9 kN) and the absence of significant plastic deformation expected within the applied stress range. The material response under load was governed by Hooke’s Law, expressed in three dimensions as provided in Eq. 6:

6 $$\:{\varvec{\sigma\:}}_{\varvec{i}\varvec{j}}={\varvec{C}}_{\varvec{i}\varvec{j}\varvec{k}\varvec{l}}\times\:{\varvec{\epsilon\:}}_{\varvec{k}\varvec{l}}$$

where:

- σ_ij_ is the stress tensor.
- ε_kl_ is the strain tensor.
- C_ijkl_ ​is the fourth-order elasticity tensor, which, for isotropic materials, depends only on Young’s modulus (E) and Poisson’s ratio (ν).

Boundary conditions were applied to simulate real-world loading and support. The bottom surface of each lattice was fixed in all directions to represent an anchored structure, while the top surface was subjected to three different loadings (3 kN, 6 kN, 9 kN) in the negative z-direction. The applied force was uniformly distributed over the top surface area, calculated as provided in Eq. 7:

7 $$\:\varvec{F}=\varvec{P}\cdot\:\varvec{A}$$

where P is the pressure and A is the surface area.

**Fig. 4:**
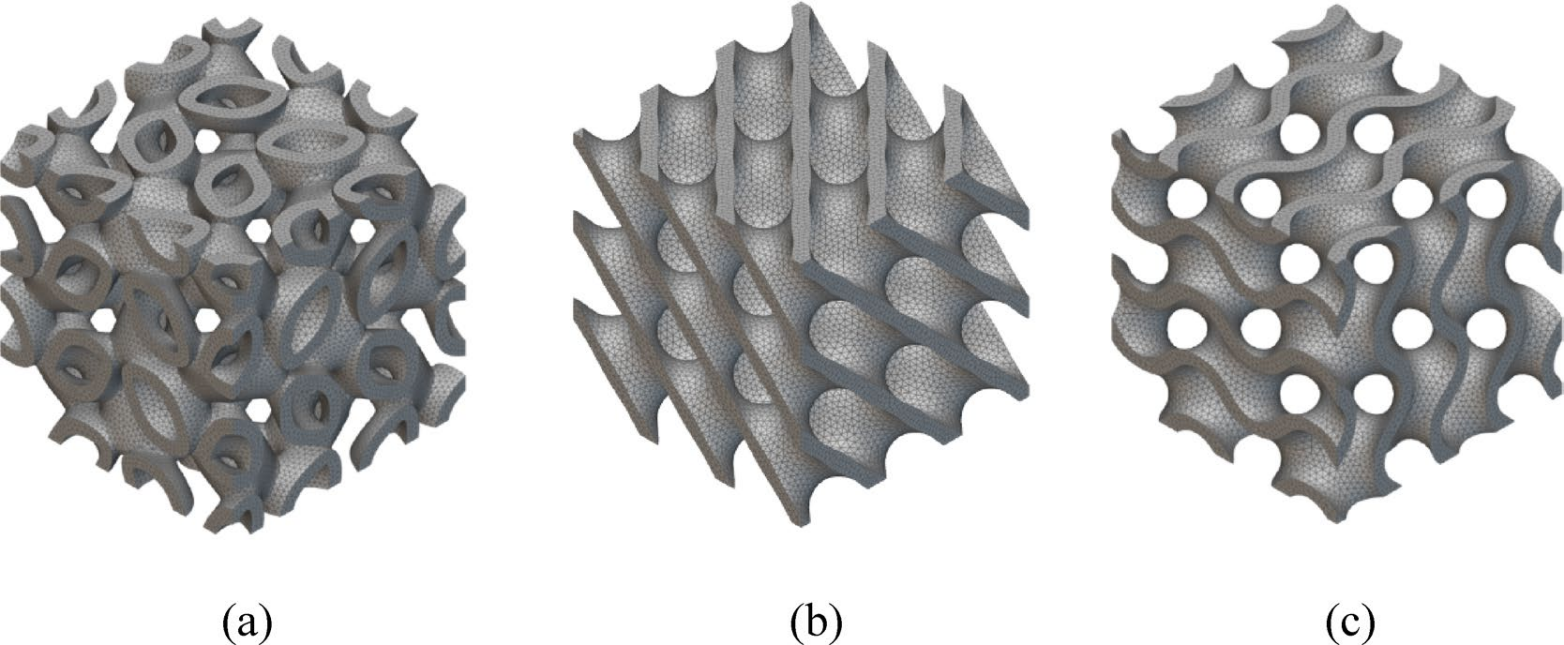
Tetrahedral meshing of TPMS lattices modelled using nTop (version 5.17.2; https://www.ntop.com/); (**a**) Lidinoid; (**b**) Diamond; (**c**) Gyroid.

Meshing was performed with tetrahedral elements (Fig. 4), ensuring a mesh size of 0.5 mm for the struts and nodes. A finer mesh was applied at critical regions, such as junctions and areas of high stress, to capture accurate results. Mesh quality was checked to avoid distorted elements, and a mesh convergence study was conducted to ensure accuracy, with further refinement showing negligible changes in results. A mesh convergence study was carried out using three different mesh sizes: 0.75 mm, 0.5 mm, and 0.3 mm. The maximum displacement for the Gyroid lattice under 9 kN load were tracked as the representative output responses. The change in displacement between 0.75 mm and 0.5 mm meshes was 6.2%, whereas the difference between 0.5 mm and 0.3 mm was only 2.8%. Hence, 0.5 mm was selected as the optimal mesh size, balancing accuracy and computational time. This confirmed that the results are mesh independent within acceptable engineering tolerance.

A linear static solver was used to analyze the lattice behavior under applied loads. The displacement δ for each lattice under loading was calculated using Hooke’s Law for the individual struts as provided in Eq. 8:

8 $$\:\varvec{\delta\:}=\frac{\varvec{F}\varvec{L}}{\varvec{A}\varvec{E}}$$

where F is the applied force, L is the strut length, A is the strut cross-sectional area, and E is the Young’s Modulus.

## Results and discussion

This section details the experimental and computational findings i.e. displacement and strain measurements are primarily analyzed using Design of Experiments to understand the material’s response. Further, a BPANN model is developed using experimental data to predict these responses. Finally, FEA simulations are used to validate the experimental outputs, ensuring accuracy and consistency.

### Experimental analysis

**Fig. 5:**
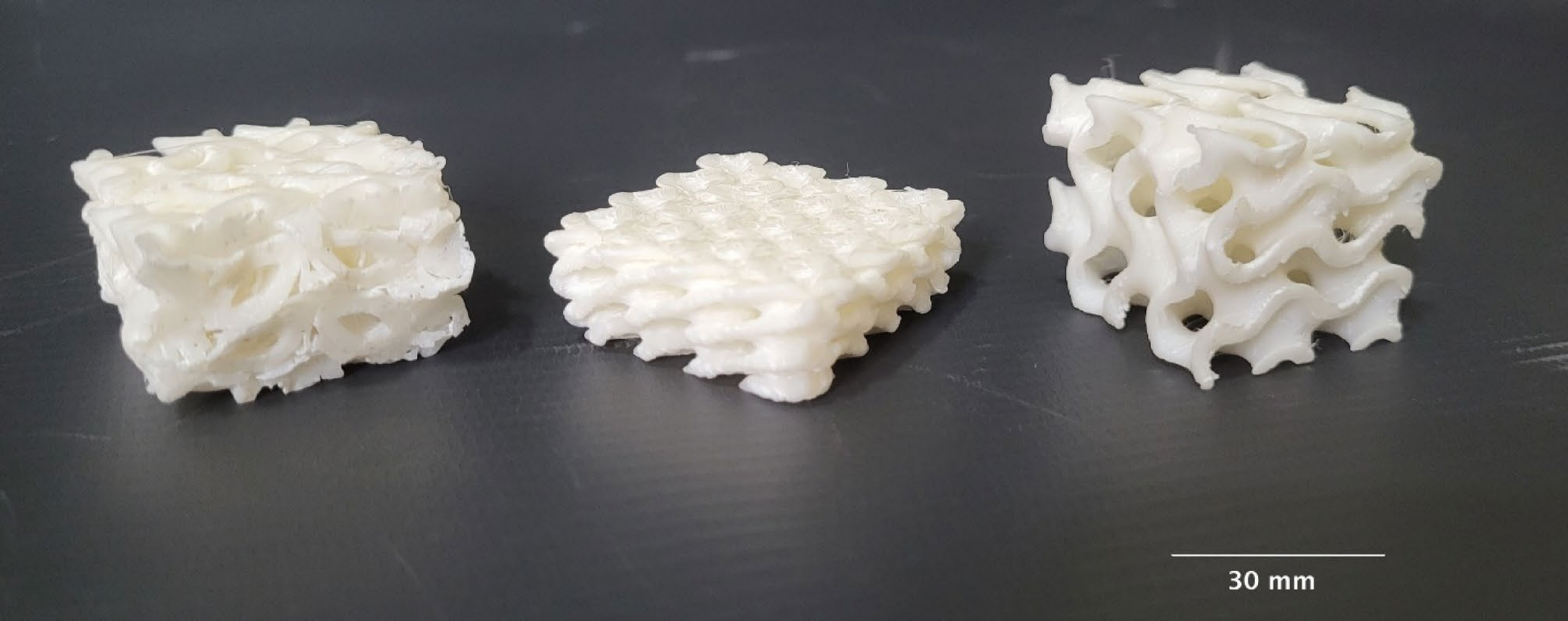
Deformed lattice of Lidinoid, Diamond, and Gyroid under compression.

The compression behavior of the three different lattice structures i.e. Lidinoid, Diamond, and Gyroid was evaluated at varying wall thicknesses (1 mm, 1.5 mm, and 2 mm), and the load-displacement curves were analyzed to understand the mechanical response of each topology. Figure 5 presents the deformed lattices after compression analysis. From the experimental results (Table 3) it was observed that the Lidinoid lattice exhibited the highest displacement among all three structures at comparable loads, indicating greater deformability under axial compression. The Diamond structure showed intermediate displacement values, while the Gyroid lattice demonstrated the least deformation, suggesting a relatively stiffer architecture.

For all three lattices, an increase in wall thickness resulted in improved load-bearing capacity and reduced overall displacement, which is consistent with the fundamental principle that thicker walls provide greater resistance to elastic and plastic deformation. The Lidinoid, characterized by its highly contoured and intricate internal structure, failed at a load of approximately 10 kN, showing significant buckling and collapse of strut elements. In contrast, the Diamond lattice with its more angular framework sustained up to 12 kN before exhibiting signs of structural failure, which was characterized by localized crushing and shear deformation.

Among the tested geometries, the Gyroid lattice displayed the highest strength, withstanding a compressive load of up to 13.5 kN before reaching its yield point. The yield behavior was distinctly identified by a plateau or flattening in the load-displacement curve, a typical characteristic indicating the onset of plastic deformation. At this stage, the structure no longer offers significant resistance to further loading, and strain continues to increase with little or no increase in applied load. This is indicative of yielding in cellular structures where local collapse or densification begins to dominate the deformation mode.

The higher yield strength and reduced displacement in the Gyroid lattice can be attributed to its triply periodic minimal surface (TPMS) geometry, which provides isotropic mechanical properties and efficient load distribution. The interconnected network of smooth surfaces in the Gyroid allows for uniform stress transfer and minimizes stress concentrations, thereby enhancing its load-carrying capacity. Conversely, Lidinoid’s convoluted curvature may promote early buckling under compressive loading, leading to premature failure.

Overall, the experimental results indicate a clear correlation between lattice topology, wall thickness, and compressive performance. The Gyroid structure, particularly at 2 mm wall thickness, emerged as the most promising candidate for load-bearing applications due to its superior strength and delayed onset of yield.

**Table 3:** Experimental results.

Sl. No.	Geometry Type	Wall Thickness (mm)	Load (kN)	Displacement (mm)	Strain × 10^−2^
1	Lidinoid	1.0	3	2.84	9.47
2	Lidinoid	1.0	6	2.95	9.84
3	Lidinoid	1.0	9	3.17	10.57
4	Lidinoid	1.5	3	2.56	8.54
5	Lidinoid	1.5	6	2.63	8.77
6	Lidinoid	1.5	9	2.70	9.0
7	Lidinoid	2.0	3	2.24	7.47
8	Lidinoid	2.0	6	2.31	7.7
9	Lidinoid	2.0	9	2.42	8.07
10	Diamond	1.0	3	1.86	6.2
11	Diamond	1.0	6	1.94	6.47
12	Diamond	1.0	9	2.06	6.87
13	Diamond	1.5	3	1.05	3.5
14	Diamond	1.5	6	1.12	3.74
15	Diamond	1.5	9	1.24	4.14
16	Diamond	2.0	3	0.82	2.74
17	Diamond	2.0	6	0.96	3.2
18	Diamond	2.0	9	1.02	3.4
19	Gyroid	1.0	3	0.62	2.07
20	Gyroid	1.0	6	0.84	2.8
21	Gyroid	1.0	9	1.04	3.47
22	Gyroid	1.5	3	0.45	1.5
23	Gyroid	1.5	6	0.52	1.74
24	Gyroid	1.5	9	0.64	2.14
25	Gyroid	2.0	3	0.36	1.2
26	Gyroid	2.0	6	0.41	1.37
27	Gyroid	2.0	9	0.48	1.6

**Fig. 6:**
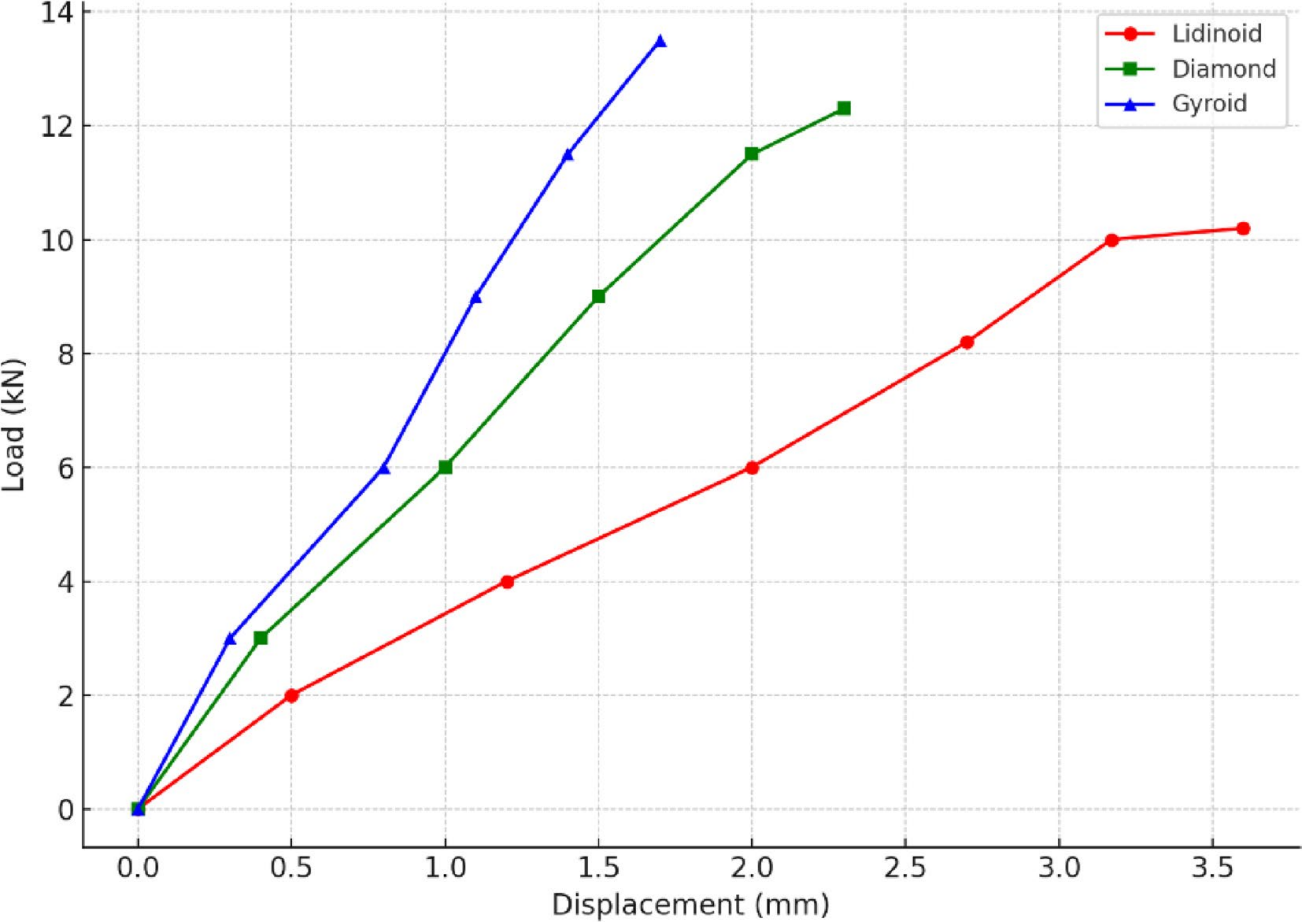
Load vs. Displacement curves up to Failure for the three TPMS scaffold geometries.

To validate the mechanical failure behavior of the TPMS scaffolds, detailed load-displacement curves were generated for each geometry, as shown in Fig. 6. The Lidinoid structure exhibited a steep increase in load-bearing capacity up to 10.2 kN, beyond which the curve plateaued, indicating structural instability. The sharp drop in slope corresponds to global buckling of the thin struts and layer collapse, particularly near the mid-height of the scaffold. This behavior aligns with Euler-type buckling typically observed in thin-walled cellular structures. Post-compression visual inspection revealed kinking of vertical struts, inward folding, and delamination, affirming the onset of plastic deformation and buckling.

The Diamond lattice demonstrated a relatively more gradual curve, sustaining compressive loads up to ~ 12.3 kN. Beyond this point, the load response stabilized, signifying shear deformation and localized crushing at nodal intersections. This is attributed to the angular strut connectivity in Diamond structures, which creates stress concentration zones. Cracks propagated diagonally across the unit cells, indicating that shear failure mechanisms governed the collapse, rather than global instability.

In contrast, the Gyroid structure showed the most stable and efficient mechanical performance, sustaining a load of up to 13.5 kN. The curve demonstrated a smooth and continuous rise without abrupt transitions, reflecting progressive densification rather than sudden failure. The displacement remained within 1.7 mm, confirming high stiffness and resistance to both buckling and shear. The Gyroid’s continuous surface topology and uniform stress distribution likely delayed localized damage, allowing it to absorb higher energy under compression.

These observations confirm that the failure modes are strongly geometry-dependent, with Lidinoid prone to early global buckling, Diamond failing by shear, and Gyroid showing a ductile-like, energy-absorbing collapse behavior. The failure data not only support the mechanical ranking of the geometries but also provide justification for the selection of 9 kN as the upper bound for safe testing to avoid total failure in experimental samples.

#### Displacement

**Fig. 7:**
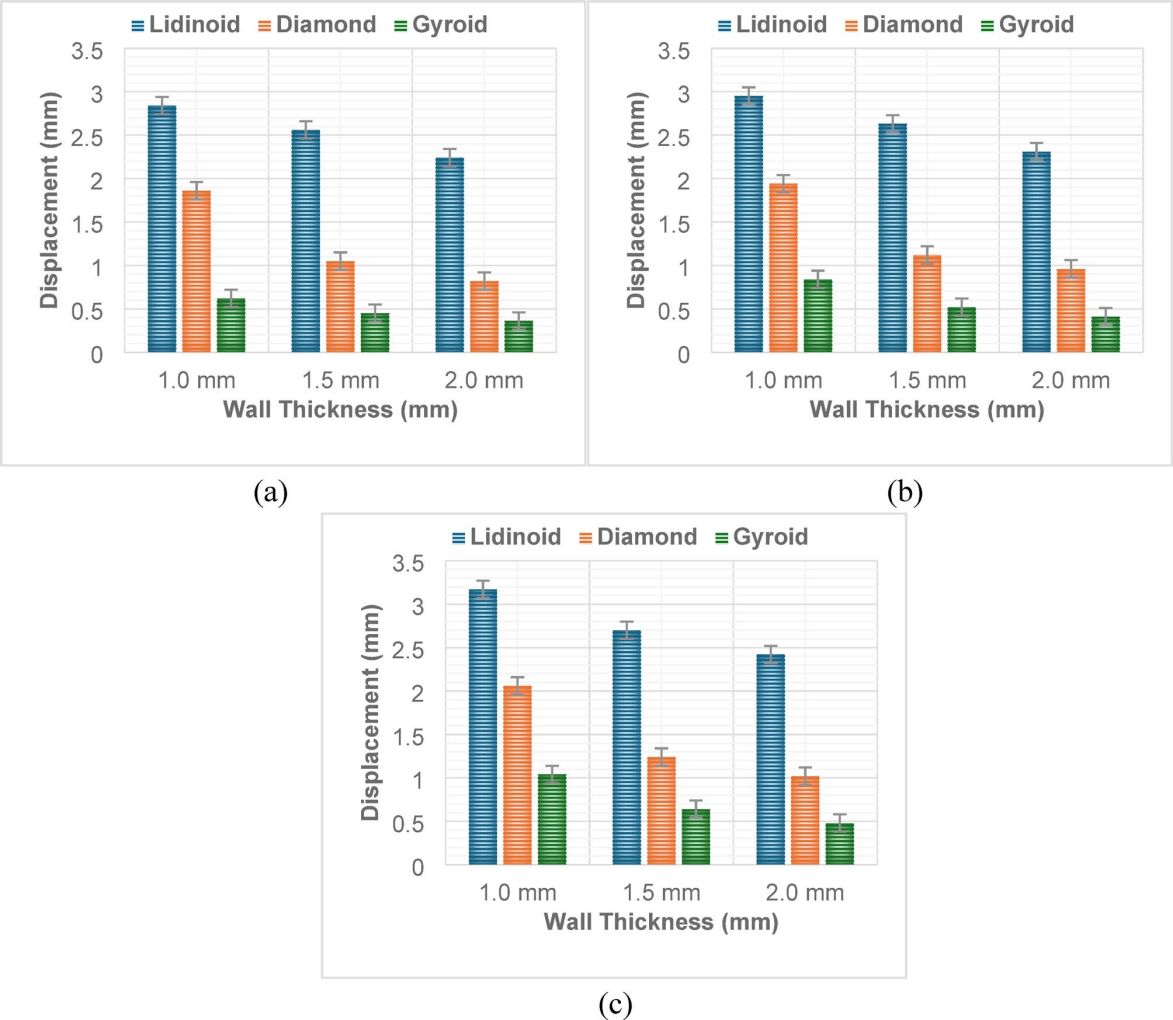
Variation of displacement along with wall thickness for (**a**) Load – 3kN; (**b**) Load – 6kN; (**c**) Load – 9kN.

The displacement behavior of lattice structures under compression is significantly influenced by the geometry type, wall thickness, and applied load. Among the three geometries (Fig. 7), the Gyroid lattices consistently exhibited the highest displacement values at a fixed wall thickness of 1.0 mm and across all load levels, followed by Diamond and then Lidinoid. For instance, at a 9 kN load, the displacement for Gyroid was 1.04 mm, while Diamond and Lidinoid recorded 2.06 mm and 3.17 mm, respectively. This trend suggests that Gyroid structures are more compliant due to their continuous and smooth surface-based architecture, which, although beneficial for isotropic behavior, provides less resistance to axial deformation^11^. In contrast, the Diamond geometry, with its strut-based framework and angular node intersections, offers moderate stiffness but undergoes noticeable localized deformation under increasing load^12,13^. Lidinoid, being a triply periodic minimal surface (TPMS) like Gyroid, demonstrates a more complex and tortuous internal path that promotes efficient stress distribution, resulting in the lowest displacement values and thus indicating higher stiffness and energy absorption capacity^14^.

Wall thickness plays a crucial role in modulating displacement across all geometries. An increase in wall thickness from 1.0 mm to 2.0 mm results in a substantial reduction in displacement under the same loading conditions. For example, in Lidinoid lattices subjected to 9 kN, displacement decreased from 3.17 mm at 1.0 mm thickness to 2.42 mm at 2.0 mm. A similar trend is evident in Diamond and Gyroid geometries, confirming the inverse relationship between wall thickness and displacement. Thicker walls enhance the effective cross-sectional area and the second moment of inertia of the lattice structure, thereby offering greater resistance to bending, buckling, and axial deformation. As expected, displacement also increases with the increase in compressive load, indicative of elastic and elasto-plastic behavior. For every geometry and wall thickness combination, the displacement rises steadily from 3 kN to 9 kN, although some non-linear behavior is observed, particularly in Diamond lattices with higher wall thicknesses. This anomaly, where displacement reduces with increasing load, indicates the onset of structural densification, where collapsing parts of the lattice interlock and resist further deformation.

**Fig. 8:**
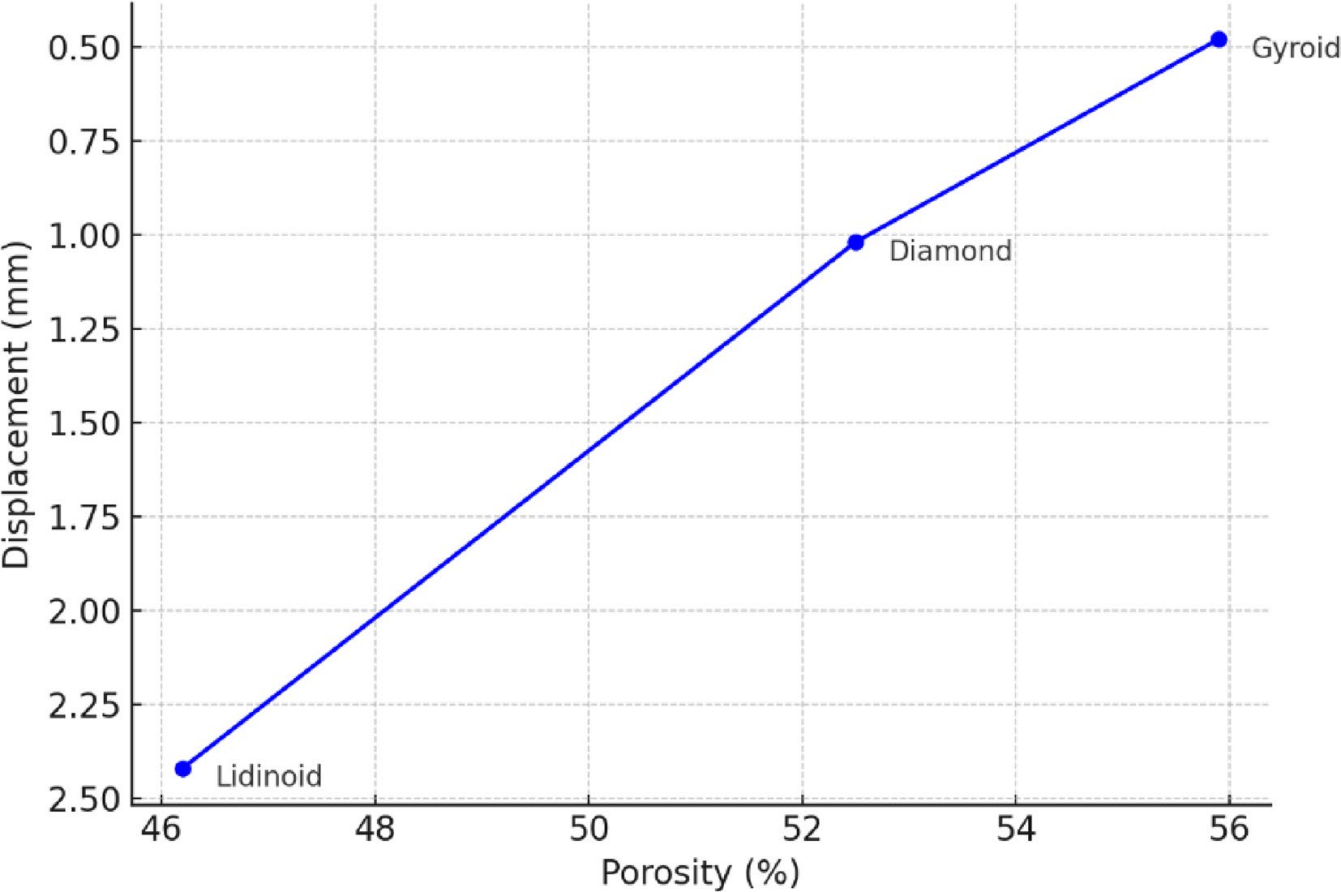
Porosity vs. Displacement for the three TPMS lattice structures at 2.0 mm wall thickness under 9 kN load.

From Fig. 8 it can be deduced that, Gyroid, despite having the highest porosity (~ 56%), showed the least deformation, confirming that geometric continuity and stress distribution play a stronger role than porosity alone. Lidinoid, with the lowest porosity (~ 46%), deformed more, suggesting that surface complexity without optimal load path distribution compromises mechanical integrity.

The interdependence of geometry, wall thickness, and load is crucial in defining the overall mechanical response. While geometry dictates the intrinsic stiffness and deformation behavior, wall thickness determines the robustness of the load-bearing paths, and applied load governs the extent of their utilization. For instance, the Gyroid structure with a thickness of 1.0 mm is highly deformable under 9 kN, but increasing the wall thickness to 2.0 mm significantly reduces its displacement, nearly matching that of the Lidinoid. This demonstrates how increased wall thickness compensates for geometric compliance. In summary, Lidinoid lattices are stiffer and more deformation-resistant due to their complex TPMS architecture, Diamond lattices provide moderate stiffness with a strut-dominated framework, and Gyroid lattices, although structurally isotropic, are the most compliant under load due to their smooth, continuous walls.

The Analysis of Variance (ANOVA) (Table 4) results reveal a statistically significant influence of all three main factors i.e. Geometry (A), Wall Thickness (B), and Load (C), on the displacement behavior of the lattice structures. Among them, the geometry type exhibits the most dominant effect, accounting for 84.38% of the total variation in displacement, as evidenced by its high F-value (2269.52) and a p-value of 0.000, indicating strong statistical significance. Wall thickness contributes 11.37% to the variation, with a corresponding F-value of 305.88, also highly significant (*p* = 0.000). Though comparatively less influential, the applied load (C) still plays a meaningful role (1.56% contribution) with an F-value of 41.87 and a p-value well below 0.05, suggesting that all three factors independently affect the mechanical response of the lattices.

Interaction effects were also evaluated, revealing that the interaction between geometry and wall thickness (A×B) is significant, contributing 1.92% to the total variance and showing a strong F-value of 25.81. The geometry-load interaction (A×C) accounts for 0.59%, and though it has a lower F-value of 7.99, it remains statistically significant (*p* = 0.007). Conversely, the interaction between wall thickness and load (B×C) contributes minimally (0.03%) and is not statistically significant (*p* = 0.083), suggesting that these two factors act largely independently in influencing displacement. The residual error (R.E.) accounts for 0.15% of the total variance, indicating that the model has captured nearly all systematic variation. This is further validated by the high coefficient of determination (R² = 98.2%) and adjusted R² (94.0%), demonstrating excellent model fit and predictive accuracy.

**Table 4:** Analysis of variance for displacement (mm).

Source	DF	Seq SS	Adj SS	Adj MS	F	*P*	*P*%
**A**	2	806.728	806.728	403.364	2269.52	0.000	84.3819786
**B**	2	108.728	108.728	54.364	305.88	0.000	11.3727102
**C**	2	14.882	14.882	7.441	41.87	0.000	1.55662455
**A×B**	4	18.351	18.351	4.588	25.81	0.000	1.91947433
**A×C**	4	5.678	5.678	1.419	7.99	0.007	0.59390634
**B×C**	4	0.254	0.254	0.063	0.36	0.083	0.02656784
**R.E.**	8	1.422	1.422	0.178			0.14873808
**Total**	26	956.043					100
S = 1.194; R^2^ = 98.2%; R^2^(adj) = 94.0%							

Where, A – Geometry Type; B – Wall Thickness; C – Load; R.E. – Residual Error.

**Fig. 9:**
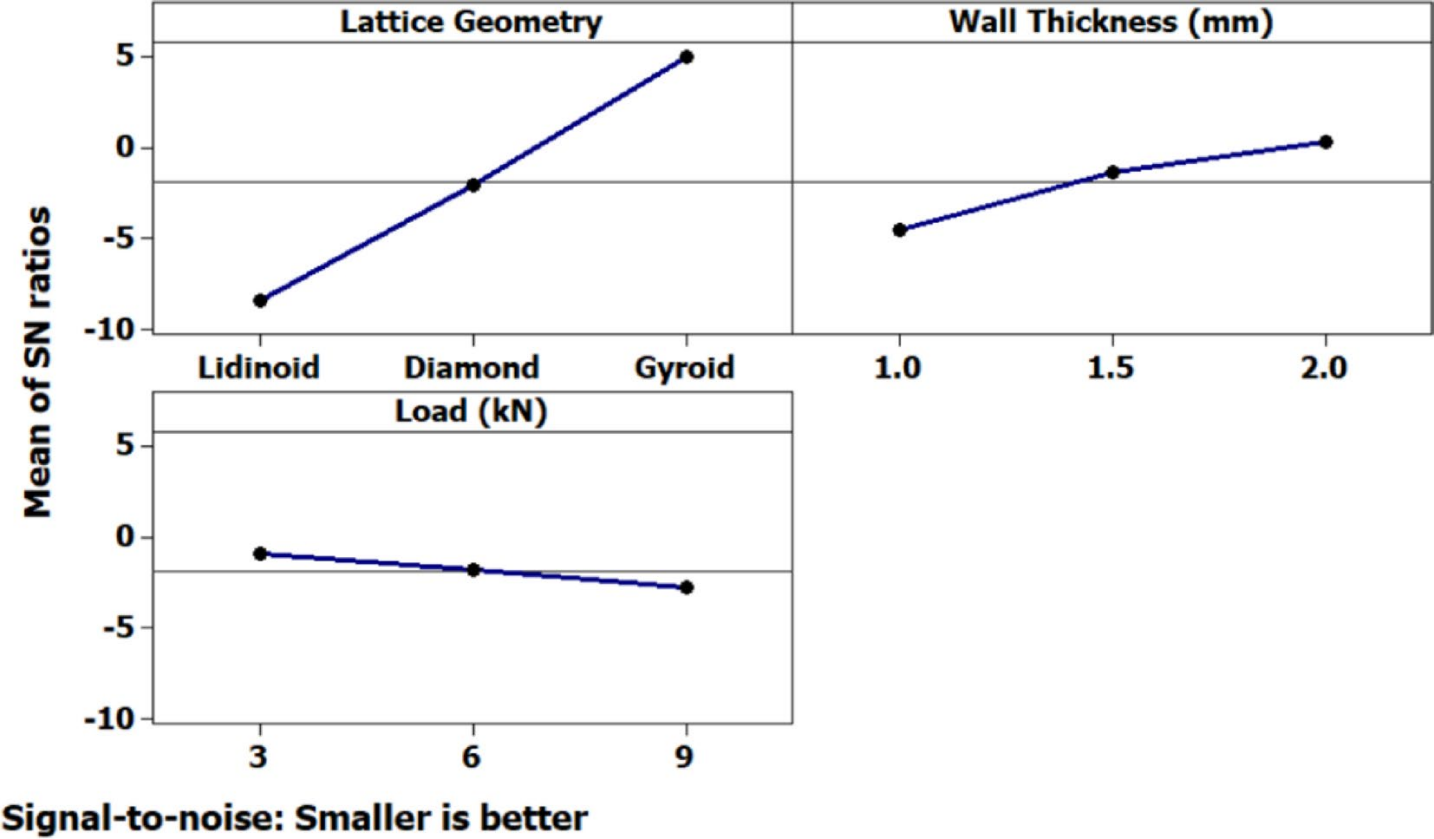
Main effects plot for displacement.

The main effects plot for the signal-to-noise (S/N) ratio (Fig. 9), based on the “smaller-is-better” criterion, reveals the relative influence of each input parameter on the measured output. Among the three lattice geometries tested, the Gyroid structure exhibited the highest (least negative) S/N ratio, indicating superior performance, whereas the Lidinoid structure demonstrated the lowest S/N value, suggesting relatively poor performance. An increase in wall thickness from 1.0 mm to 2.0 mm resulted in a consistent improvement in S/N ratio, signifying that thicker walls contribute to enhanced resistance under loading. Conversely, increasing the applied load from 3 kN to 5 kN led to a marginal decline in S/N ratio, implying that higher loads negatively impact the mechanical response.

**Fig. 10:**
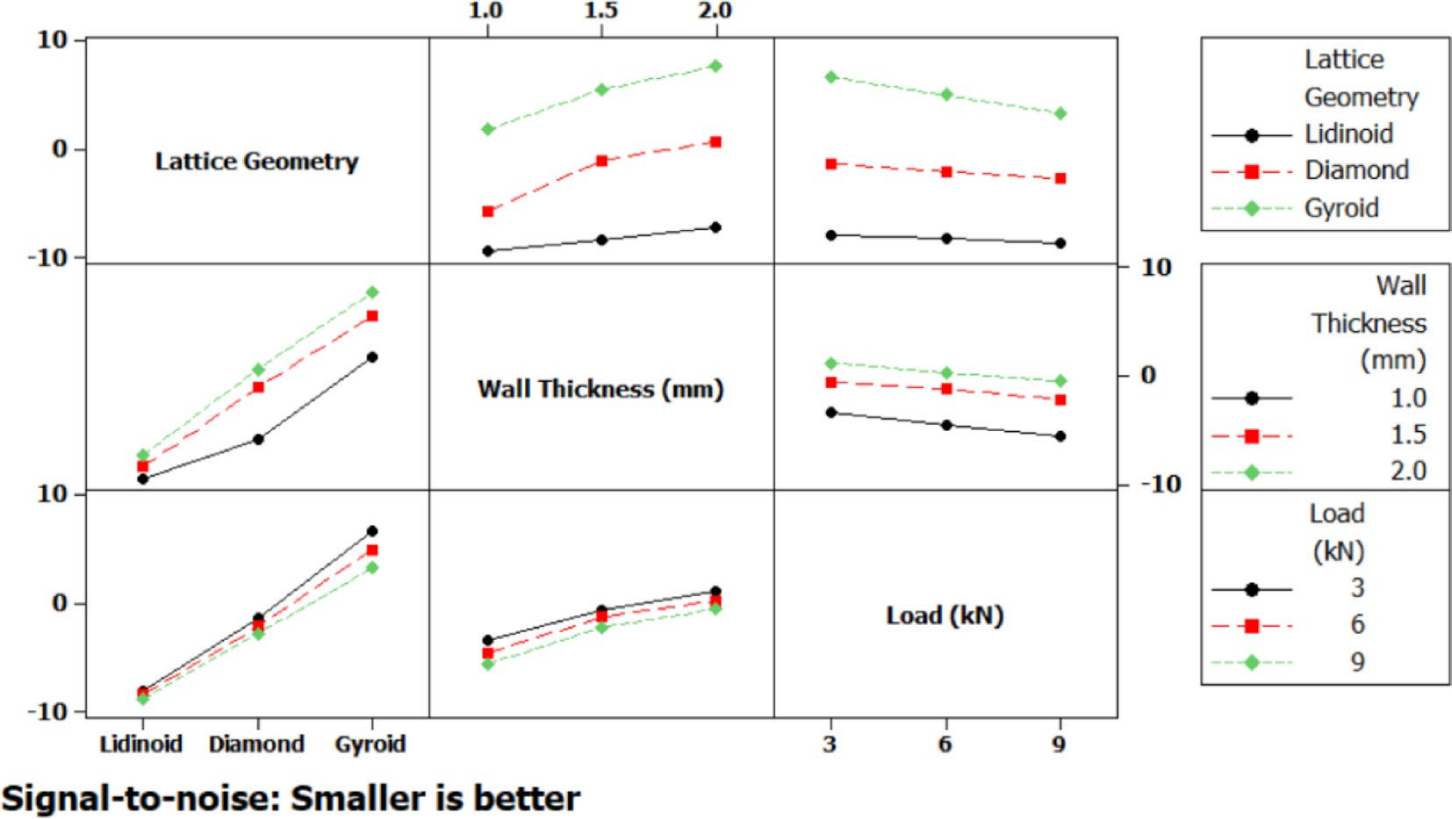
Interaction plot for displacement.

Interaction plots (Fig. 10) further clarified the combined effects of the process parameters on performance. A strong interaction was observed between lattice geometry and wall thickness, wherein the Gyroid structure particularly benefited from an increase in wall thickness, while the Lidinoid structure showed relatively poorer outcomes regardless of wall thickness. A notable interaction between wall thickness and applied load was also present, indicating that the benefits of increased wall thickness may be diminished at higher loads. Interactions between lattice geometry and load appeared less significant, although the Lidinoid geometry consistently underperformed across all load levels. These interactions underscore the importance of optimizing combinations of parameters rather than considering them in isolation.

**Fig. 11:**
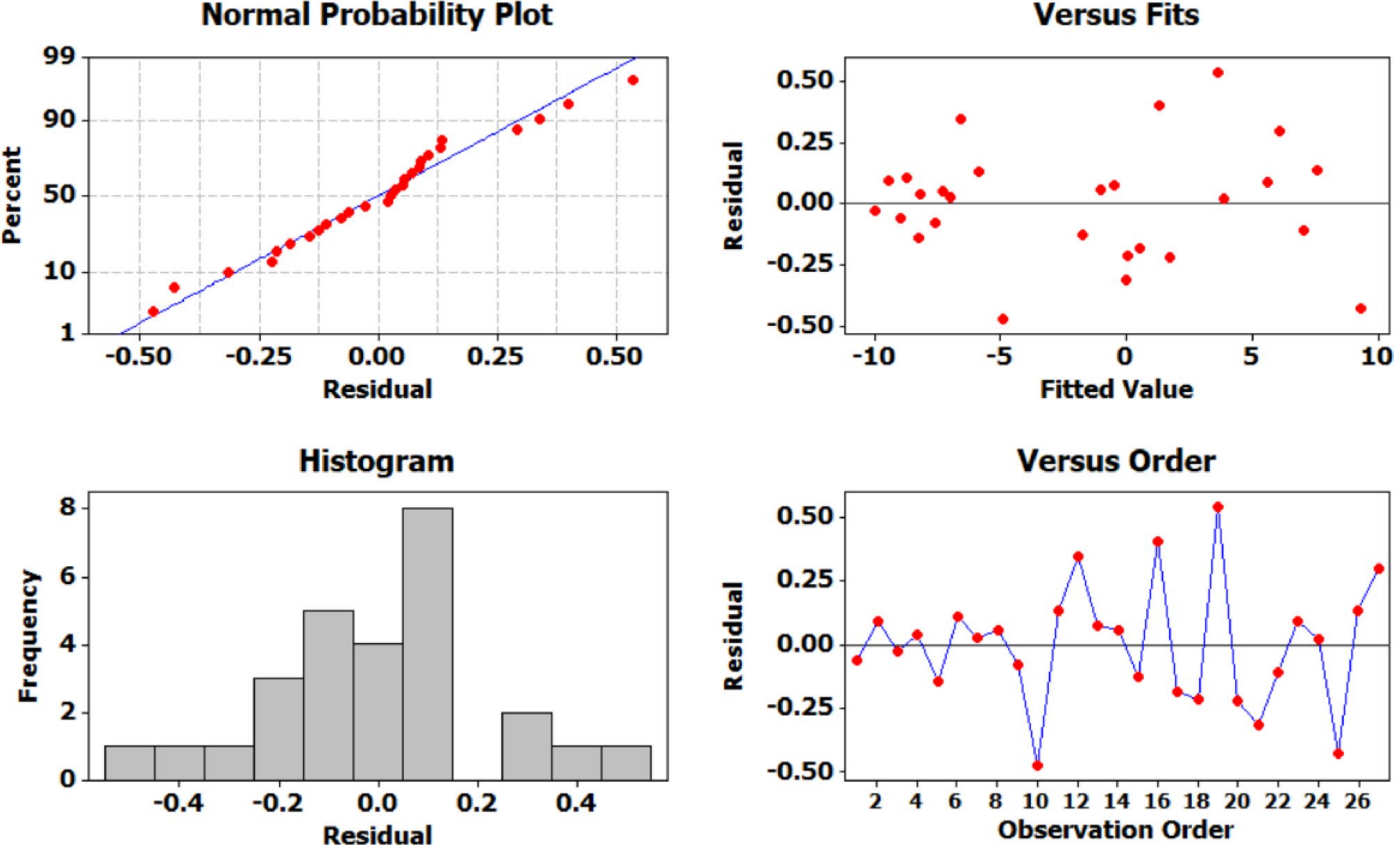
Residual plots for displacement.

The adequacy of the Taguchi-based model was validated through residual plots (Fig. 11). The normal probability plot displayed residuals that closely followed a straight line, confirming the assumption of normality. The residuals versus fits plot showed a random scatter without any apparent patterns, indicating homoscedasticity and confirming that the variance remained constant across the range of fitted values. Additionally, the histogram of residuals was symmetric and centered around zero, further supporting normal distribution. The residuals versus order plot exhibited no discernible trends or systematic variation, verifying the independence of residuals. Collectively, these diagnostic plots affirmed the validity of the experimental model and the reliability of the results.

**Fig. 12:**
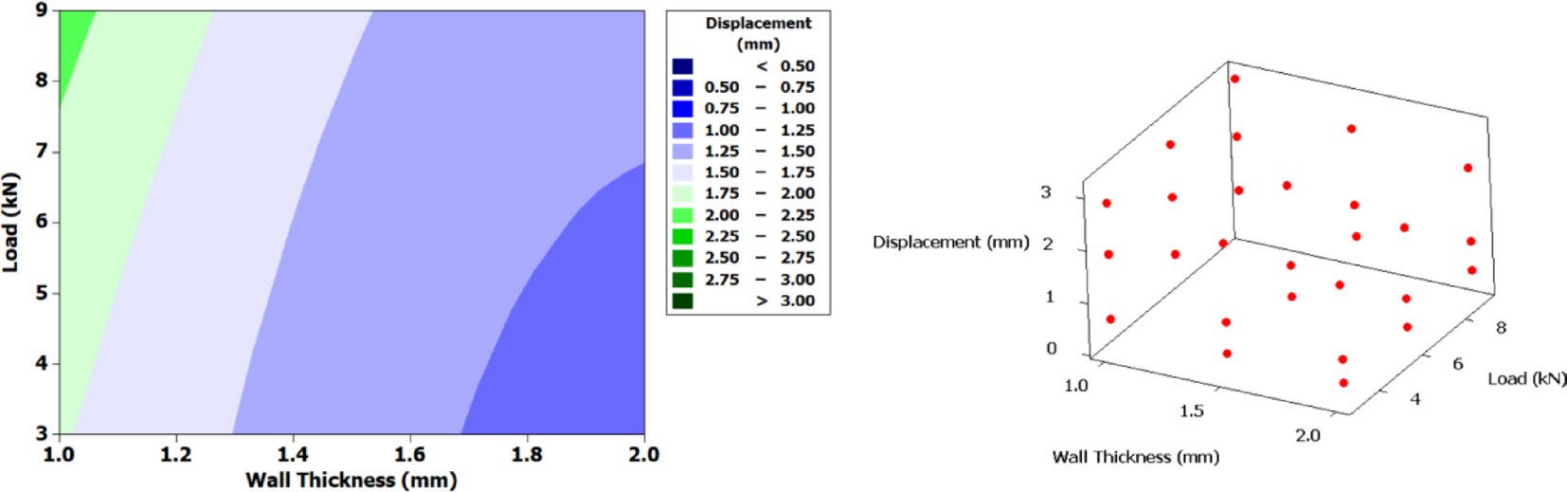
Contour plot and 3D scatter plot for displacement.

Figure 12 presents contour plot along with 3D scatter plot for displacement. A clear gradient in displacement is observed as wall thickness increases, particularly under higher loads. Thinner walls (1.0–1.2 mm) exhibit significantly larger displacements, especially when subjected to loads above 7 kN, with values exceeding 3 mm. In contrast, thicker walls (closer to 2 mm) demonstrate minimal displacement (< 0.5 mm) under comparable loading conditions. This trend implies that increasing the wall thickness enhances structural stiffness, thereby reducing deformation even under elevated mechanical loading. The clustering of points along the upper displacement range at lower thicknesses and higher loads reinforces the observations from the contour plot. Conversely, data points with lower displacement are predominantly located at regions with increased wall thickness and lower load application.

#### Strain

**Fig. 13:**
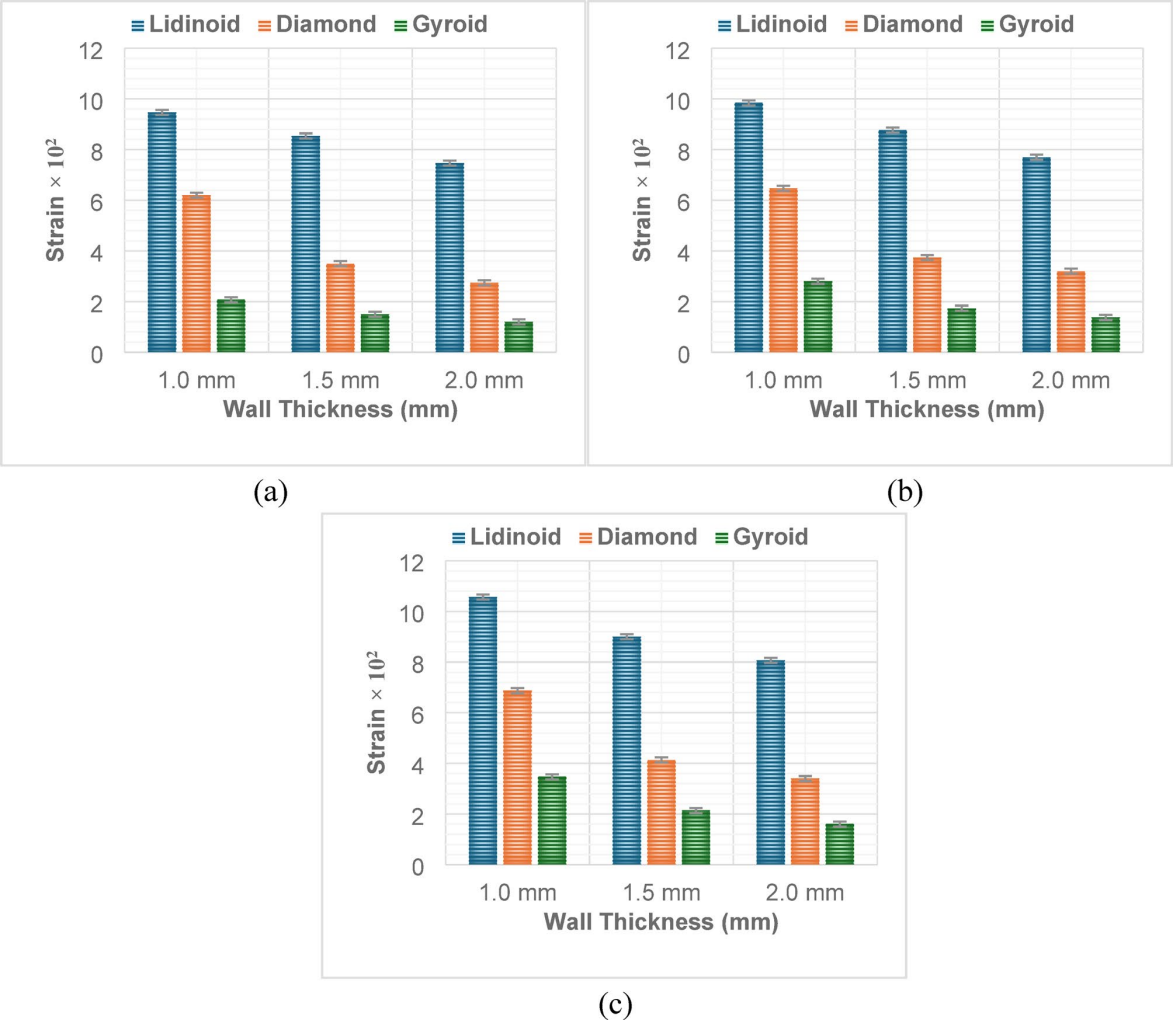
Variation of strain along with wall thickness for (**a**) Load – 3kN; (**b**) Load – 6kN; (**c**) Load – 9kN.

The strain behavior of lattice structures under varying load conditions and wall thicknesses reveals a strong dependency on the geometry type. Among the three geometries investigated, distinct trends in strain evolution were observed (Fig. 13).

For the Lidinoid structure, strain increased consistently with increasing load for a fixed wall thickness. At 1.0 mm thickness, strain rose from 9.47 × 10⁻² at 3 kN to 10.57 × 10⁻² at 9 kN. However, an inverse trend was observed with wall thickness; at constant load, increasing wall thickness led to lower strain values. For instance, under 9 kN load, strain decreased from 10.57 × 10⁻² at 1.0 mm to 8.07 × 10⁻² at 2.0 mm. This indicates that thinner Lidinoid structures deform more under load due to their relatively lower stiffness.

In contrast, the Diamond geometry exhibited significantly lower strain values overall, suggesting greater stiffness. Similar to the Lidinoid type, strain increased with loading for each fixed wall thickness. However, the absolute values of strain were much lower. For example, at 1.5 mm wall thickness, strain values were 3.5 × 10⁻² at 3 kN and increased to 4.14 × 10⁻² at 9 kN. Again, thicker structures exhibited reduced strain, with the strain dropping to just 3.4 × 10⁻² at 2.0 mm thickness and 9 kN load. Interestingly, Diamond structures appeared to reach a saturation or diminishing return in strain beyond 1.5 mm thickness, especially under higher loading.

The Gyroid structures showed the lowest strain among all geometries for a given wall thickness and load. At 1.0 mm thickness, strain increased from 2.07 × 10⁻² at 3 kN to 3.47 × 10⁻² at 9 kN, showing the expected load-induced rise in deformation. However, as wall thickness increased, strain dropped sharply. At 2.0 mm thickness and 9 kN load, the strain was only 1.6 × 10⁻². This indicates that Gyroid structures possess the best load-bearing capability among the tested geometries, offering minimal deformation even under maximum load.

In general, the strain was found to be highest in Lidinoid, intermediate in Diamond, and lowest in Gyroid structures, under identical input conditions. Wall thickness demonstrated a strong negative correlation with strain, while applied load showed a positive correlation. The combined effect of geometry, wall thickness, and load significantly influences the deformation characteristics of the structures. Specifically, thinner Lidinoid structures under high load conditions represent the most compliant configuration, while thicker Gyroid structures represent the stiffest design.

The ANOVA results summarized in Table 5 reveal that the Geometry Type (A) has the most significant influence on strain, accounting for 84.38% of the total variation, with a very high F-value of 2333.06 and a P-value of 0.000, indicating a statistically significant effect. The Wall Thickness (B) is the next most influential parameter, contributing 11.38% to the strain variation, also with a highly significant P-value (0.000). Load (C), while statistically significant (*P* = 0.000), contributed only 1.56%, suggesting that although load has a direct impact on strain, its effect is relatively minor when compared to geometry and wall thickness.

Among the interaction effects, the Geometry × Wall Thickness (A×B) interaction contributed 1.92%, with a significant F-value of 26.50, signifying that the combination of geometry type and wall thickness jointly influences strain response meaningfully. The Geometry × Load (A×C) interaction also showed statistical significance (*P* = 0.006), although it contributed a smaller 0.60% of the total variation. In contrast, the Wall Thickness × Load (B×C) interaction was found to be statistically insignificant (*P* = 0.833), with a negligible contribution of 0.03%, indicating minimal synergistic influence between these two parameters on strain.

The model demonstrated excellent statistical reliability with a coefficient of determination (R²) of 98.2% and an adjusted R² of 94.0%, confirming that the model explains the vast majority of the variability in strain. The residual error was very low (0.14%), further validating the adequacy and precision of the experimental model. Overall, the findings highlight that strain in lattice structures is predominantly governed by the geometry of the unit cell, followed by wall thickness, while load plays a secondary yet significant role, and only specific interactions among parameters have measurable effects.

**Table 5:** Analysis of variance for Strain.

Source	DF	Seq SS	Adj SS	Adj MS	F	*P*	*P*%
**A**	2	805.725	805.725	402.862	2333.06	0.000	84.3760897
**B**	2	108.639	108.639	54.320	314.58	0.000	11.3767526
**C**	2	14.902	14.902	7.451	43.15	0.000	1.56054794
**A×B**	4	18.307	18.307	4.577	26.50	0.000	1.91712194
**A×C**	4	5.720	5.720	1.430	8.28	0.006	0.59900243
**B×C**	4	0.246	0.246	0.061	0.36	0.833	0.02576129
**R.E.**	8	1.381	1.381	0.173			0.14461929
**Total**	26	954.921	954.921				100
S = 1.194; R^2^ = 98.2%; R^2^(adj) = 94.0%							

Where, A – Geometry Type; B – Wall Thickness; C – Load; R.E. – Residual Error.

**Fig. 14:**
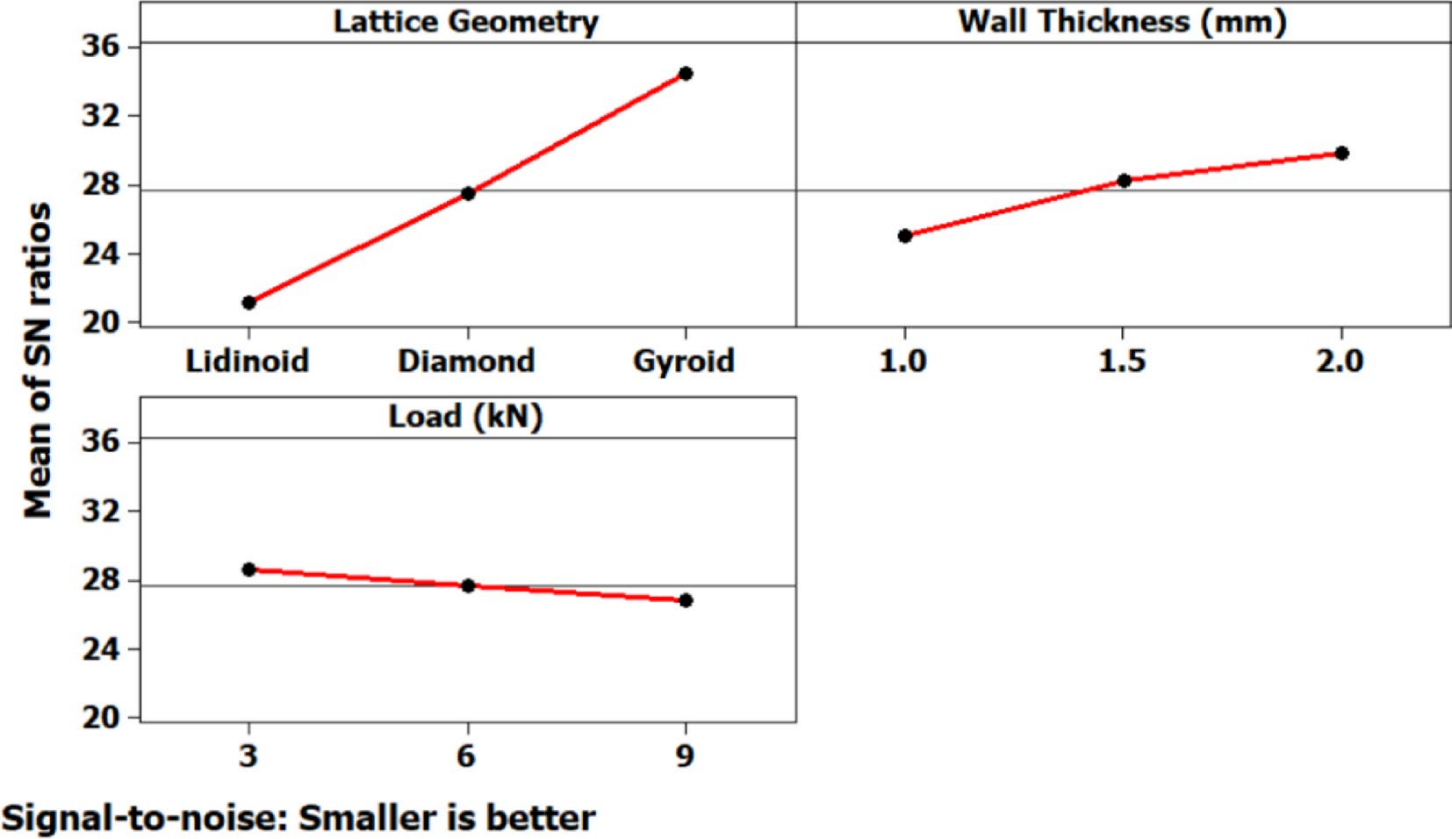
Main effects plot for strain.

The main effects plot for the signal-to-noise (S/N) ratio (Fig. 14) reveals the individual influence of each input factor i.e. lattice geometry, wall thickness, and compressive load on the strain response. Among these, lattice geometry exhibits the most prominent effect. The gyroid structure shows the highest S/N ratio, indicating the least amount of strain under compression, followed by the diamond structure. In contrast, the lidinoid geometry results in the lowest S/N ratio, signifying higher strain, and thus, a less favorable response in terms of deformation resistance. Wall thickness also significantly affects the strain behavior, where increasing the thickness from 1.0 mm to 2.0 mm leads to an upward trend in the S/N ratio, suggesting a substantial reduction in strain due to enhanced structural rigidity. On the other hand, compressive load demonstrates a milder influence; as the load increases, a slight decline in the S/N ratio is observed, implying a corresponding but moderate rise in strain.

**Fig. 15:**
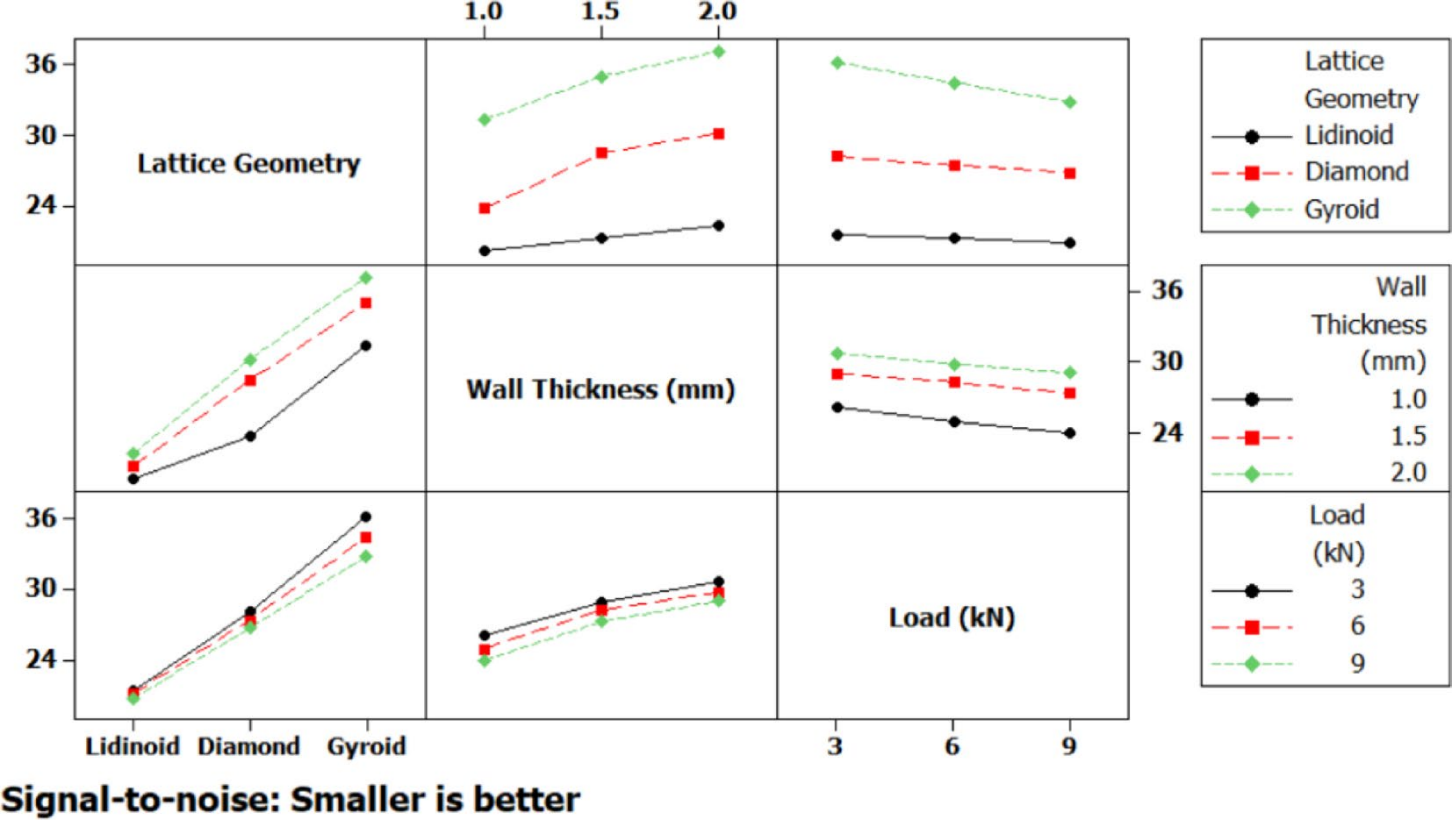
Interaction plot for strain. .

The interaction plot (Fig. 15) illustrates the combined effects of factor pairs on the strain response, shedding light on how the relationship between variables modifies the strain behavior beyond individual contributions. The most prominent interaction is observed between lattice geometry and wall thickness. For instance, the gyroid structure shows a steep increase in the S/N ratio with increasing wall thickness, indicating a pronounced improvement in strain resistance. In contrast, the Lidinoid structure displays consistently low S/N ratios regardless of thickness, signifying that increasing thickness does not significantly improve its strain performance. A mild interaction is noticed between geometry and load, where the Lidinoid structure displays a clear trend of increasing strain with higher loads, while the diamond and gyroid geometries show relatively stable behavior across different loading conditions. The interaction between wall thickness and load appears to be negligible, as represented by nearly parallel lines in the plot. This suggests that the effect of compressive load on strain is largely independent of the wall thickness.

**Fig. 16:**
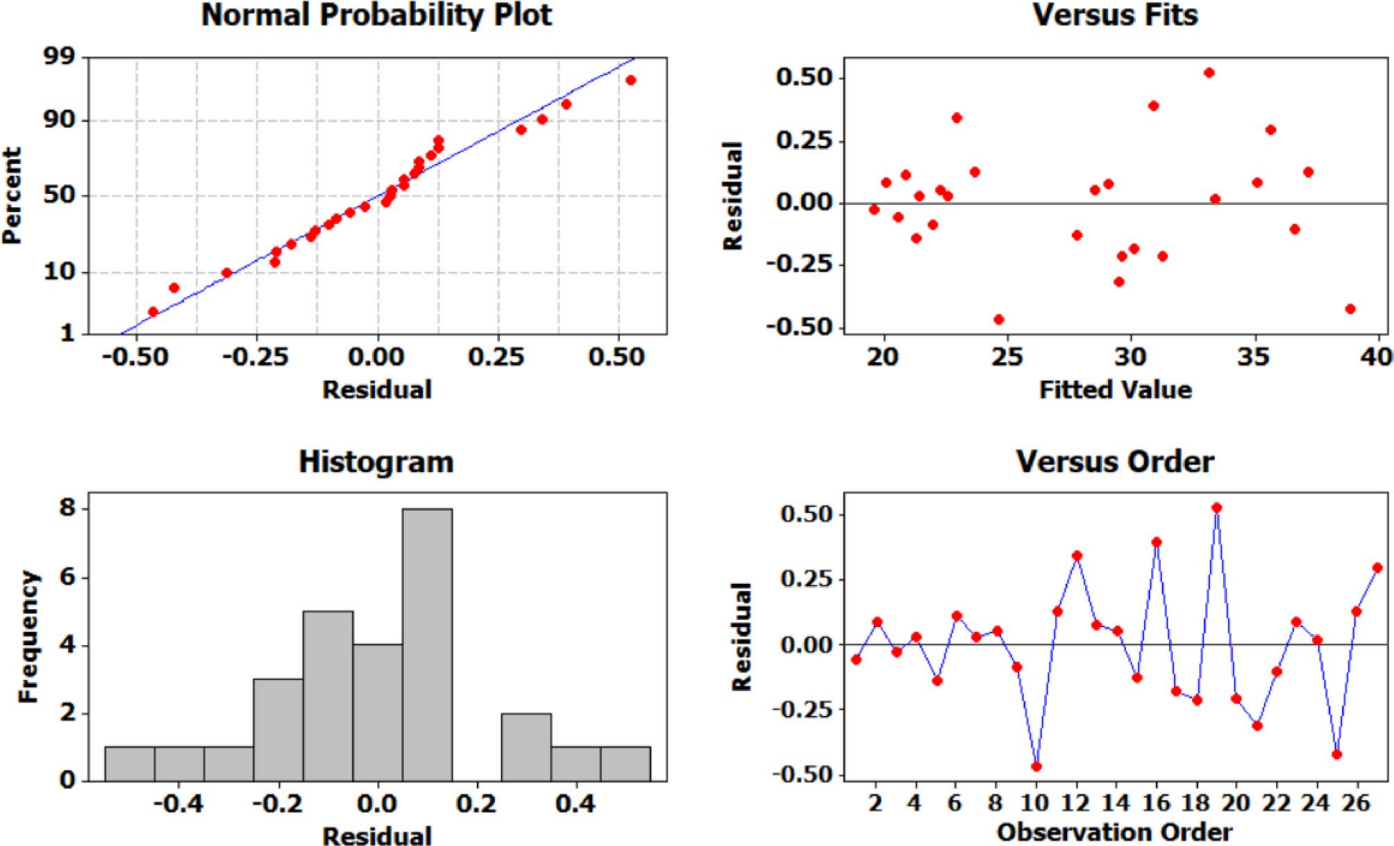
Residual plots for strain.

The residual analysis (Fig. 16) confirms the adequacy and reliability of the regression model used to predict strain behavior. The normal probability plot of the residuals displays a near-linear distribution of data points, validating the assumption of normality in the residuals. The plot of residuals versus fitted values shows a random scatter without any discernible patterns, indicating homoscedasticity i.e., constant variance across the range of predicted values. The histogram of residuals further supports this observation, showing a bell-shaped distribution consistent with normal behavior. Additionally, the plot of residuals versus observation order lacks any noticeable trend or cyclic pattern, suggesting that the residuals are uncorrelated and randomly distributed.

**Fig. 17:**
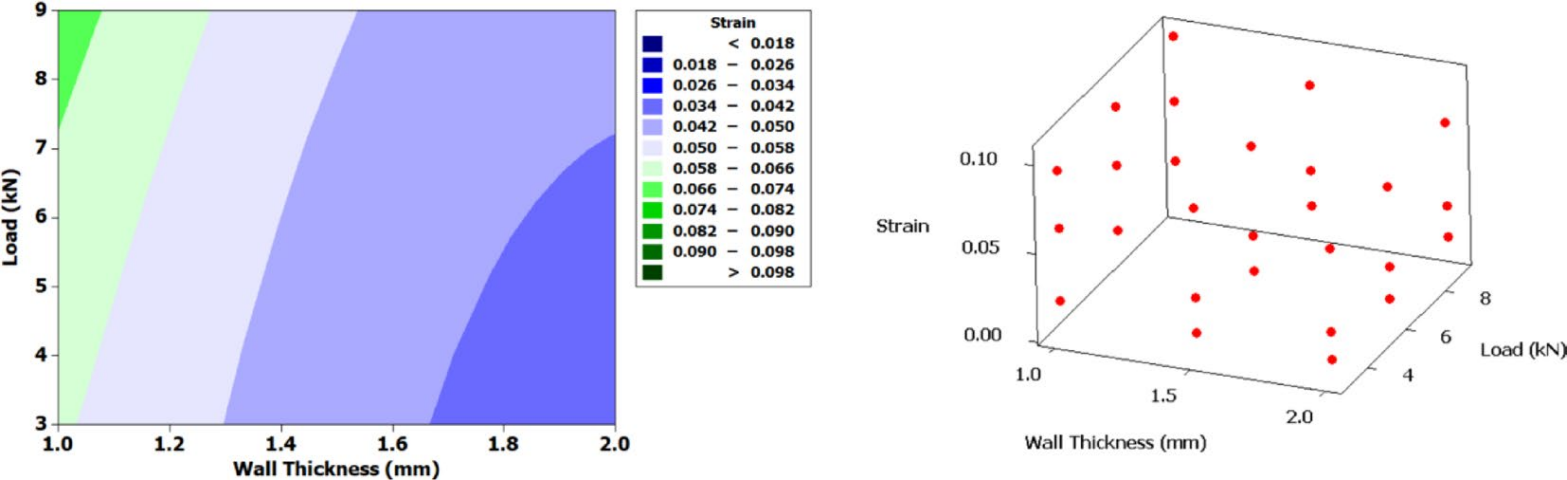
Contour plot and 3D scatter plot for strain.

Figure 17 presents the contour plot and 3D scatter plot for strain. A clear gradient in strain is observed as wall thickness increases, particularly under higher loads. Thinner walls (1.0–1.2 mm) exhibit significantly larger strain, especially when subjected to loads above 7 kN, with values exceeding 0.098. In contrast, thicker walls (closer to 2 mm) demonstrate minimal strain (< 0.026) under comparable loading conditions.

This trend implies that increasing the wall thickness enhances structural stiffness, thereby reducing deformation even under elevated mechanical loading. The clustering of points along the upper strain range at lower thicknesses and higher loads reinforces the observations from the contour plot. Conversely, data points with lower strain are predominantly located at regions with increased wall thickness and lower load application.

### Back-propagation artificial neural network prediction modelling

In this study, the performance of Back-Propagation Artificial Neural Networks (BPANN) was evaluated for predicting displacement and strain across different geometries, wall thicknesses, and load conditions.

The comparison of activation functions (Table 6) revealed that ReLU outperformed Tanh and Sigmoid, yielding the lowest Root Mean Squared Error (RMSE) and Mean Absolute Error (MAE) for both displacement and strain predictions. Specifically, ReLU showed RMSE values of 0.845 mm for displacement and 0.072 × 10⁻^2^ for strain, with R² values of 0.972 and 0.961, respectively, indicating a strong predictive capability. In contrast, the Sigmoid function exhibited the highest errors, with RMSE of 1.345 mm for displacement and 0.102 × 10⁻^2^ for strain, alongside a lower R² score of 0.923 for displacement and 0.901 for strain.

**Table 6:** Comparison of activation functions.

Activation Function	Output Variable	RMSE	*R*²	MAE
**ReLU**	Displacement (mm)	0.845	0.972	0.690
	Strain (×10⁻^2^)	0.072	0.961	0.055
**Tanh**	Displacement (mm)	1.103	0.951	0.862
	Strain (×10⁻^2^)	0.089	0.940	0.070
**Sigmoid**	Displacement (mm)	1.345	0.923	1.095
	Strain (×10⁻^2^)	0.102	0.901	0.087

The BPANN model was trained on 18 samples (Table 7) and tested on 9 (Table 8), yielding excellent results on the testing set with R² scores of 0.9991 for displacement and 0.9954 for strain. The RMSE values for displacement and strain (Table 9) were 0.145 mm and 0.075 × 10⁻^2^ respectively, showcasing the model’s high accuracy. Furthermore, the Mean Absolute Percentage Error (MAPE) was remarkably low, particularly for displacement at 0.79%, indicating the model’s robust generalization ability. Table 10 presents the performance of the BPANN model on testing data. The maximum absolute errors for displacement and strain stress were 0.178 mm and 0.062 × 10⁻^2^, further supporting the reliability of the model predictions.

**Table 7:** Training set.

Sl. No.	Geometry Type	Wall Thickness (mm)	Load (kN)	Displacement (mm)	Strain × 10^2^
1	Lidinoid	1.0	6	2.95	9.84
2	Lidinoid	1.0	9	3.17	10.57
3	Lidinoid	1.5	3	2.56	8.54
4	Lidinoid	1.5	9	2.70	9.0
5	Lidinoid	2.0	3	2.24	7.47
6	Lidinoid	2.0	6	2.31	7.7
7	Diamond	1.0	6	1.94	6.47
8	Diamond	1.0	9	2.06	6.87
9	Diamond	1.5	3	1.05	3.5
10	Diamond	1.5	9	1.24	4.14
11	Diamond	2.0	3	0.82	2.74
12	Diamond	2.0	6	0.96	3.2
13	Gyroid	1.0	6	0.84	2.8
14	Gyroid	1.0	9	1.04	3.47
15	Gyroid	1.5	3	0.45	1.5
16	Gyroid	1.5	9	0.64	2.14
17	Gyroid	2.0	3	0.36	1.2
18	Gyroid	2.0	6	0.41	1.37

**Table 8:** Testing set.

Geometry Type	Wall Thickness (mm)	Load (kN)	Displacement (mm)	BPANN prediction	Error %	Strain × 10^2^	BPANN prediction	Error %
Lidinoid	1.0	3	2.84	2.745	3.345	9.47	9.546	0.802
Lidinoid	1.5	6	2.63	2.672	1.596	8.77	8.723	0.535
Lidinoid	2.0	9	2.42	2.387	1.363	8.07	8.127	0.706
Diamond	1.0	3	1.86	1.964	5.591	6.2	6.275	1.209
Diamond	1.5	6	1.12	1.132	1.0714	3.74	3.863	3.288
Diamond	2.0	9	1.02	1.067	4.607	3.4	3.267	3.911
Gyroid	1.0	3	0.62	0.648	4.516	2.07	2.031	1.884
Gyroid	1.5	6	0.52	0.541	4.038	1.74	1.765	1.436
Gyroid	2.0	9	0.48	0.476	0.833	1.6	1.538	3.875
Total Error (%)					1.52			1.96

**Table 9:** Summary of BPANN architecture, training settings, and performance metrics.

Parameter	BPANN Model Details
Model Type	Back-Propagation Artificial Neural Network (BPANN)
Input Features	Geometry Type (One-Hot), Wall Thickness (mm), Load (kN)
Output Targets	Displacement (mm), Strain
Total Data Samples	27
Training Samples	18
Testing Samples	9
Activation Function (Hidden Layer)	ReLU
Activation Function (Output Layer)	Linear
Number of Hidden Layers	1
Number of Neurons	12
Loss Function	Mean Squared Error (MSE)
Optimizer	Adam
Learning Rate	0.01
Epochs	1000
Batch Size	4
Train-Test Split Ratio	2:1 (18 training, 9 testing)

**Table 10:** Model performance on testing data (*n* = 9).

Metric	Displacement (mm)	Strain
R² Score	0.9991	0.9954
RMSE	0.145	0.075
MAE	0.126	0.062
MAPE	0.79%	4.03%
Max Absolute Error	0.178	0.062
Explained Variance	0.9992	0.9956

### Finite element analysis

**Fig. 18:**
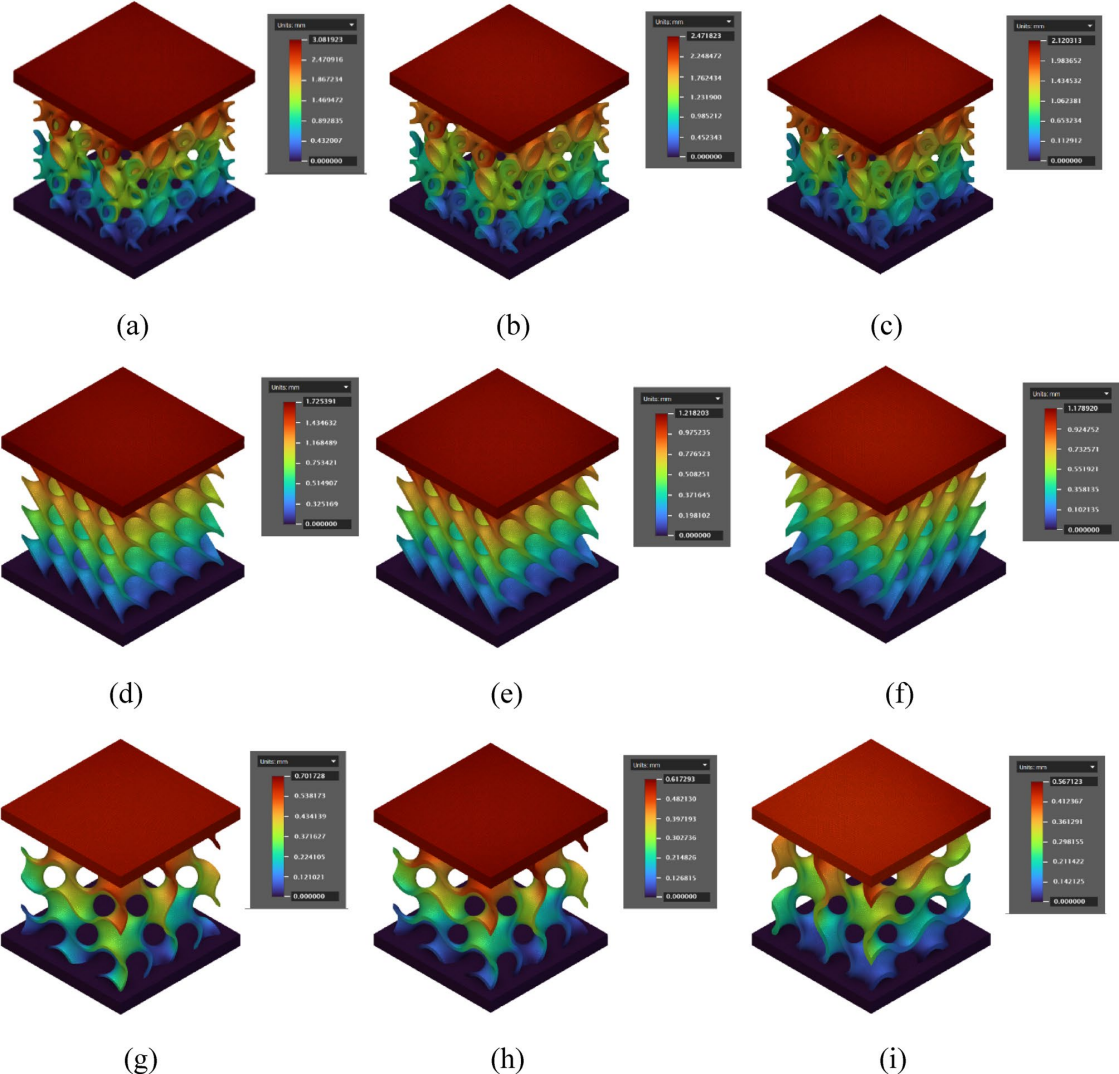
Finite element displacement analysis of various lattices under different wall thickness and loading conditions simulated using nTop (version 5.17.2; https://www.ntop.com/); (**a**) Lidinoid 1 mm 3kN; (**b**) Lidinoid 1.5 mm 6kN; (**c**) Lidinoid 2 mm 9kN; (**d**) Diamond 1 mm 3kN; (**e**) Diamond 1.5 mm 6kN; (**f**) Diamond 2 mm 9kN; (**g**) Gyroid 1 mm 3kN; (**h**) Gyroid 1.5 mm 6kN; (**i**) Gyroid 2 mm 9kN.

The finite element analysis (FEA) results for all three lattices (Fig. 18) exhibited a consistent trend in line with the experimental observations, indicating a strong correlation between numerical predictions and physical behavior under compression. The maximum displacements obtained from FEA simulations closely matched the experimentally measured values, with minor variations likely attributed to practical factors such as surface imperfections, fabrication tolerances, and slight deviations in boundary condition application.

In Lidinoid structure with a wall thickness of 1 mm, the maximum displacement predicted by FEA was approximately 3.08 mm (Fig. 18a), while the experimental value was 2.84 mm. Similarly, the Diamond (Fig. 18d) and Gyroid (Fig. 18g) structures with the same wall thickness exhibited maximum displacements of 1.72 mm and 0.70 mm in FEA, compared to 1.86 mm and 0.62 mm experimentally. This consistency between experimental and simulated data confirms the accuracy and reliability of the FEA model in capturing the global deformation response of the structures under compressive loads.

A detailed examination of the FEA results further reveals the displacement distribution across different vertical levels within the lattice structures. The displacement values gradually increased from the bottom, which was fixed in all simulations and hence showed zero displacement, to the top surface, where the compressive load was applied. This smooth and progressive increase in displacement from the base to the top indicates that the deformation primarily occurred in a linear-elastic manner, without abrupt changes or localized failures. This pattern of displacement is theoretically governed by the fundamentals of elasticity and boundary condition constraints in vertical compression. Since the bottom of the lattice is fixed, it remains stationary, acting as a rigid support. When a compressive load is applied to the top, the lattice elements deform progressively along the load path. The magnitude of displacement at any given height within the structure depends on the local stiffness, wall thickness, and geometry of the unit cell.

In all three lattice types, the displacement at intermediate levels followed a smoothly rising trend, with no sharp jumps or irregularities, suggesting that the stress and strain were distributed evenly across the structure. For instance, in the Lidinoid structure with 1 mm wall thickness, the displacement gradually increased from zero at the bottom to around 0.43 mm at the first level, 0.89 mm at the second, 1.47 mm at the third, and reached the maximum of 3.08 mm at the top. A similar distribution was observed in the other geometries. This uniform distribution can be explained through classical beam and shell theories, where the strain accumulation increases with distance from the fixed support due to the cumulative nature of deformation. Moreover, in periodic lattice structures, each successive layer experiences not only the externally applied load but also the reaction transmitted through the layers beneath, resulting in increasing displacement as one moves upward.

The observed deformation pattern also highlights the effect of wall thickness on overall stiffness. As wall thickness increases, the structural members offer higher resistance to compression, thereby reducing the magnitude of displacement throughout the height of the lattice. For example, the Lidinoid structure at 2 mm wall thickness showed a top displacement of only 2.12 mm, compared to 3.08 mm for the 1 mm variant. Similar reductions were observed in Diamond and Gyroid geometries. This inverse relationship between wall thickness and displacement further corroborates the analytical understanding of stiffness, where increased cross-sectional area enhances load-bearing capacity and reduces elastic deformation.

The progression of displacement from zero at the bottom to maximum at the top is a direct outcome of applied boundary conditions and elastic behavior under axial loading. The theoretical principles of stress transfer, stiffness variation, and boundary constraints adequately explain the observed deformation characteristics in the simulated models. The study confirms that lattice geometry and wall thickness play a critical role in governing the deformation mechanics, and FEA serves as a reliable tool to predict such behaviors with high accuracy.

**Table 11:** Comparison of FEA simulation results with experimental results.

Geometry Type	Wall Thickness (mm)	Load (kN)	Experimental Displacement (mm)	FEA Simulation	Error %
Lidinoid	1.0	3	2.84	3.08	8.45
Lidinoid	1.5	6	2.63	2.47	6.08
Lidinoid	2.0	9	2.42	2.12	12.39
Diamond	1.0	3	1.86	1.73	6.98
Diamond	1.5	6	1.12	1.21	8.03
Diamond	2.0	9	1.02	1.17	14.7
Gyroid	1.0	3	0.62	0.7	12.9
Gyroid	1.5	6	0.52	0.61	17.3
Gyroid	2.0	9	0.48	0.56	16.66

Table 11 presents a comparative analysis between the finite element analysis (FEA) simulation results and experimental displacement values under varying wall thicknesses and loading conditions for three scaffold geometries. For all geometries, the displacement values predicted by FEA show reasonable agreement with experimental results, with error percentages ranging from 6.08 to 17.3%. Among the geometries, the Lidinoid structure consistently exhibited higher displacement values due to its relatively less dense internal framework, while the Gyroid structure demonstrated the least displacement under similar loading, indicating its superior mechanical stiffness.

The observed discrepancies between experimental and simulated results can be primarily attributed to dimensional inaccuracies and process-induced variations inherent in the 3D printing process. Minor deviations in wall thickness, residual stresses, surface roughness, and imperfect layer bonding during fused deposition modeling (FDM) could lead to differences in actual mechanical response compared to the idealized FEA model, which assumes perfect geometry, uniform material properties, and ideal boundary conditions.

Despite these practical deviations, the overall error remains within an acceptable range for biomedical scaffold applications, confirming that the simulation framework reliably predicts structural behavior. This validates the effectiveness of the orthogonal array-based FEA methodology used for scaffold optimization in this study.

## Conclusion

The compressive performance of PLA + triply periodic lattice structures was studied using experimental, statistical, neural network, and FEA-based methods. Geometry type had the greatest effect on mechanical behavior, contributing 84.38% to displacement and 84.37% to strain variations, as shown by ANOVA. Wall thickness and load contributed 11.37% and 1.56%, respectively, in both analyses.

Among the geometries, Gyroid lattices demonstrated the lowest displacement (0.36 mm) and lowest strain (1.2 × 10⁻²) at 3 kN and 2.0 mm thickness, confirming superior stiffness and stress distribution. Lidinoid lattices showed the highest displacement (3.17 mm) and strain (10.57 × 10⁻²) at 9 kN and 1.0 mm thickness, indicating higher deformability. Diamond lattices offered balanced performance, with 1.02 mm displacement and 3.4 × 10⁻² strain under 9 kN at 2.0 mm thickness.

An increase in wall thickness from 1.0 mm to 2.0 mm resulted in significant stiffness enhancement across all geometries. For example, Gyroid displacement reduced from 1.04 mm to 0.48 mm, and strain dropped from 3.47 × 10⁻² to 1.6 × 10⁻² under 9 kN loading.

The BPANN model predicted mechanical responses with high accuracy, achieving R² = 0.9991 (displacement) and R² = 0.9954 (strain) on the test set, with RMSE values of 0.145 mm and 0.075 × 10⁻², respectively.

FEA results matched experimental trends closely. For instance, the FEA-predicted displacement for Lidinoid (1 mm, 3 kN) was 3.08 mm, closely matching the experimental value of 2.84 mm. Displacement gradients from bottom to top surfaces confirmed linear-elastic deformation and isotropic stress propagation in Gyroid lattices.

Thus, the Gyroid geometry with 2.0 mm thickness was identified as the optimal scaffold design due to its minimal displacement and strain under all loading conditions. This study establishes a robust framework combining experimental design, FEA, and neural networks for optimizing 3D-printed bone scaffolds with high mechanical fidelity.

### Future scope

The development of a novel hybrid TPMS scaffold that integrates the most advantageous features of the Gyroid, Lidinoid, and Diamond structures holds great promise for advancing bone tissue engineering. By blending the mechanical stability of the Lidinoid, the stress distribution ability of the Gyroid, and the balanced structural response of the Diamond, a new scaffold geometry can be designed to support both load-bearing and biological performance requirements more effectively.

The output characteristics of a bone scaffold such as porosity, mechanical strength, elastic modulus, and stress distribution play a crucial role in influencing the success of tissue regeneration. An optimal scaffold must closely match the native bone in mechanical behavior to avoid stress shielding while also providing a suitable environment for cell proliferation and nutrient transport. Hence, any deviation in these output properties can negatively affect osseointegration and the long-term stability of the implant.

To achieve scaffolds with desired output characteristics, it is essential to optimize the design parameters as well as the 3D printing conditions. Parameters like infill density, build direction, and print resolution significantly impact the final geometry, surface morphology, and dimensional accuracy of the scaffold. These in turn influence how well the scaffold supports new tissue formation. Future studies should therefore focus not only on structural innovations but also on process optimization to ensure reproducibility, mechanical integrity, and biological compatibility.

## Data Availability

All data generated or analyzed during this study are included in this published article.
